# Structure-Guided Mutations in the Terminal Organelle Protein MG491 Cause Major Motility and Morphologic Alterations on *Mycoplasma genitalium*

**DOI:** 10.1371/journal.ppat.1005533

**Published:** 2016-04-15

**Authors:** Luca Martinelli, Luis García-Morales, Enrique Querol, Jaume Piñol, Ignacio Fita, Bárbara M. Calisto

**Affiliations:** 1 Instituto de Biología Molecular de Barcelona (IBMB-CSIC), Parc Científic de Barcelona, Barcelona, Spain; 2 Institut de Biotecnologia i Biomedicina and Departament de Bioquímica i Biologia Molecular, Universitat Autònoma de Barcelona, Bellaterra, Barcelona, Spain; 3 Structural Biology Group, European Synchrotron Radiation Facility, CS 40220, Grenoble, France and Instituto de Biologia Molecular e Celular (IBMC), Universidade do Porto, Porto, Portugal; Miami University, UNITED STATES

## Abstract

The emergent human pathogen *Mycoplasma genitalium*, with one of the smallest genomes among cells capable of growing in axenic cultures, presents a flask-shaped morphology due to a protrusion of the cell membrane, known as the terminal organelle, that is involved in cell adhesion and motility and is an important virulence factor of this microorganism. The terminal organelle is supported by a cytoskeleton complex of about 300 nm in length that includes three substructures: the terminal button, the rod and the wheel complex. The crystal structure of the MG491 protein, a proposed component of the wheel complex, has been determined at ~3 Å resolution. MG491 subunits are composed of a 60-residue N-terminus, a central three-helix-bundle spanning about 150 residues and a C-terminal region that appears to be quite flexible and contains the region that interacts with MG200, another key protein of the terminal organelle. The MG491 molecule is a tetramer presenting a unique organization as a dimer of asymmetric pairs of subunits. The asymmetric arrangement results in two very different intersubunit interfaces between the central three-helix-bundle domains, which correlates with the formation of only ~50% of the intersubunit disulfide bridges of the single cysteine residue found in MG491 (Cys87). Moreover, *M*. *genitalium* cells with a point mutation in the MG491 gene causing the change of Cys87 to Ser present a drastic reduction in motility (as determined by microcinematography) and important alterations in morphology (as determined by electron microscopy), while preserving normal levels of the terminal organelle proteins. Other variants of MG491, designed also according to the structural information, altered significantly the motility and/or the cell morphology. Together, these results indicate that MG491 plays a key role in the functioning, organization and stabilization of the terminal organelle.

## Author Summary

*Mycoplasma genitalium* is one of the smallest bacteria known and a common human pathogen. *M*. *genitalium* cells present a flask-shaped morphology due to the presence of a characteristic protrusion, known as the terminal organelle, that has several key biological roles. The terminal organelle allows mycoplasmas to move on solid surfaces by a distinctive type of cellular motility that is also related to pathogenicity. In the present study we have determined the first crystal structure of a terminal organelle protein (MG491). Using the structural information, we have designed and prepared several variants of this protein. *M*. *genitalium* cells expressing the variant proteins showed striking differences with respect to the unmodified mycoplasma cells. In one of the variants, we replaced a cysteine residue by a serine, which implies the exchange of just one sulfur atom by oxygen, resulting in a drastic reduction of motility and important alterations in cell morphology. Other variants changed the speed or the frequency of the movements. These results demonstrate that MG491 plays a key role in the functioning, organization and stabilization of the terminal organelle and are also a clear example of the interplay between atomic resolution details and the highest levels of cellular organization.

## Introduction

Mycoplasmas are microorganisms belonging to the class of Mollicutes (‘soft skin’) that evolved from Gram-positive bacteria by genome reduction and are characterized by the absence of a cell wall, by their small cell sizes and by their reduced biosynthetic machinery. Consequently, these microorganisms live in nature as obligate parasites depending on the uptake of essential nutrients from their hosts. Mycoplasma species vary in form and many are able to move by gliding motility [1,2]. In particular, *Mycoplasma genitalium*, one of the smallest autoreplicative microorganisms known, is a motile species belonging to the *pneumoniae* cluster of mycoplasmas. Due to its small genome, of only ~480 protein-coding genes, *M*. *genitalium* has been used as a model of minimal cell [3,4] and is the subject of intense work in systems biology research [5–7]. *M*. *genitalium* is an emergent and prevalent sexually transmitted pathogen involved in urogenital infections in humans, including non-gonococcal and non-chlamydial urethritis and inflammatory reproductive tract diseases in women. A review article on this issue described the need for an early diagnostic of the infection, which increases the risk of HIV transmission when persistent [8,9]. In addition, an intense search for novel therapeutic agents against *M*. *genitalium* has been launched as several studies revealed the existence of isolates resistant to treatments with azithromycin [10–12], indicating that there is a continuous need to search for potential drug and vaccine targets in this microorganism [11,13].

*M*. *genitalium* has a flask-shaped morphology that consists of a cell body with a protrusion of the cell membrane, called the terminal organelle, which is the scaffold for cell adhesion, division and motility, processes deeply related to infectivity. The terminal organelle is supported by a complex cytoskeleton that is formed by three main substructures: the terminal button, the rod and the wheel complex located, respectively, at the tip, the center and the rear with respect to the cell body [2,14–16]. Moreover, adhesins P110 and P140, which are very abundant at the surface of the terminal organelle, are essential for attachment to host cells, together with the accessory proteins MG218, MG312, MG317 and MG491 [17–20] A model for gliding motility in mycoplasmas from the *pneumoniae* cluster proposed a cyclic process where the rod, anchored to the wheel complex, has a central role [14,15]. According to this model in a first step, the tip of the terminal organelle binds to the substrate with the rod fully extended and, in a second step, the rod contracts, dragging the cell forward. However, we have very recently demonstrated that this model is no longer valid since *M*. *genitalium* cells remain motile in the absence of the rod element [20]. The same work also highlighted the role of P110 and P140 adhesins and of the P32 protein on promoting cell movement as previously proposed [1,21]. The wheel complex might also be involved in chromosome segregation by attaching the mycoplasma chromosome to the terminal organelle [22]. *M*. *genitalium* MG200 and MG491 proteins have been proposed as components of the terminal organelle wheel complex ultrastructure [23,24] and in agreement with this, it has recently been found that MG200 and MG491 interact with each other specifically influencing cell motility [25].

In this work, the crystal structure of MG491 was determined and found to present a unique tetrameric organization as a dimer of asymmetric pairs of subunits. The structural information guided the design of MG491 variants, which presented striking alterations in cell motility and in cell morphology demonstrating the key role played by MG491 in the organization and functioning of the terminal organelle of *M*. *genitalium*.

## Results

### Structure determination of MG491

**Fig 1:**
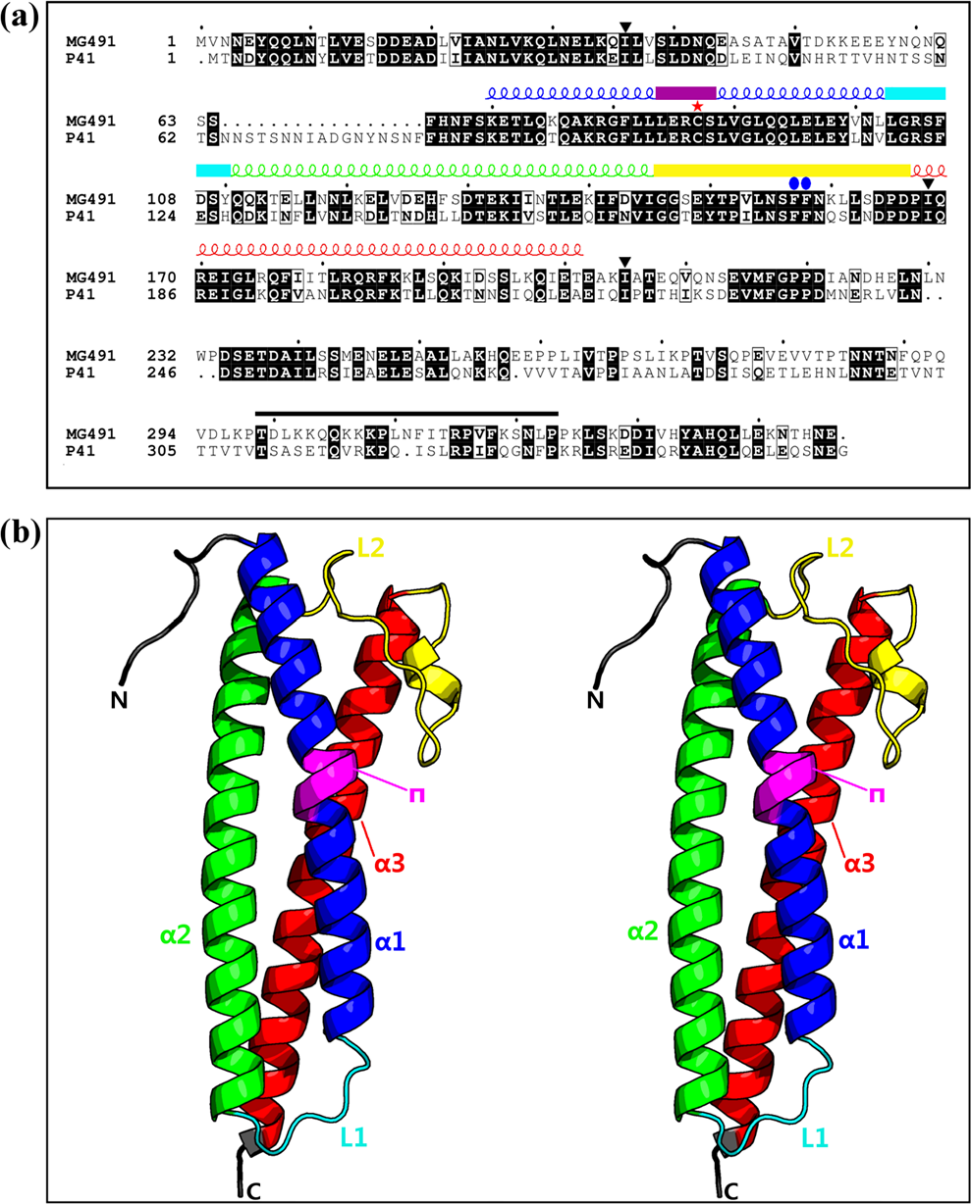
Overall structure of the MG491 monomer. (a) Sequence alignment of the *M*. *genitalium* MG491 protein with its *M*. *pneumoniae* homolog, P41. The secondary structure elements found for MG491, as defined with the Dictionary of Secondary Structure of Proteins (DSSP) algorithm [26,27], are reported above the sequence alignment and are colored as: helix α1, α2, α3 and π respectively in blue, green, red and magenta, loop L1 in cyan and loop L2 in yellow. Black solid down triangles, red asterisk and blue solid oblong dots are for residues which have been mutated into methionines (Ile36, Ile168 and Ile 205), serine (Cys87) and alanines (Phe157 and Phe158), respectively. The protein region that interacts with the terminal organelle protein MG200 is indicated with a black bar. Black boxes define conserved regions between MG491 and P41. (b) Stereoview showing the overall structure of a MG491 subunit (PDB entry code 4XNG). The protein backbone is colored according to the color-scheme in (a).

**Fig 2:**
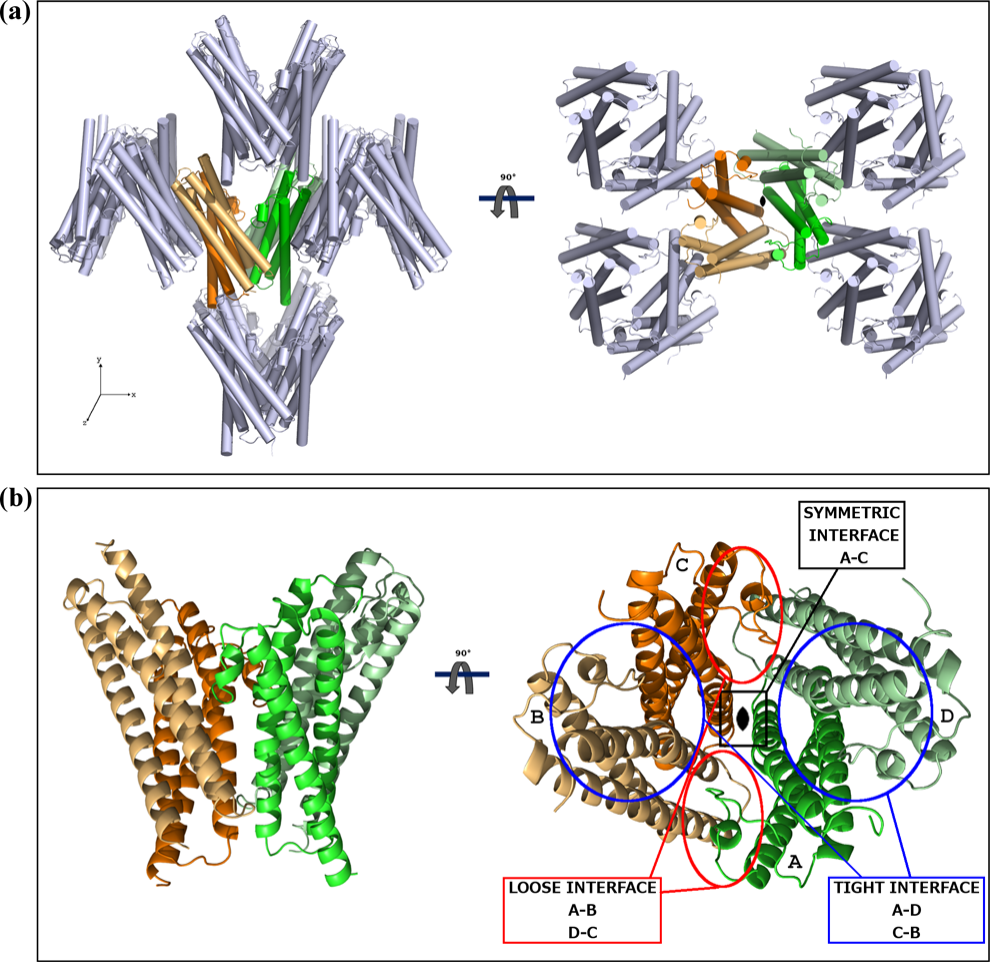
Crystal packing and the MG491 molecule. (a) Ninety degrees apart views of the MG491 crystal packing. The four subunits found in the crystal asymmetric unit (colored) present an extensive network of intersubunit interactions. The crystallographic symmetry-related subunits are displayed in grey. (b) The molecular organization of the MG491 tetramer can be described as a dimer of asymmetric pairs of subunits. The three different intersubunit interfaces found in the MG491 tetramer are indicated as symmetric (between subunits A and C), tight (between subunits A and D or, analogously, C and D) and loose (between subunits D and C or, analogously, A and B).

Crystals of the same type were obtained from both the full length MG491 protein (residues 1 to 346) and from a construct of the protein N-terminal region, MG491-Nt (residues 1 to 308), though times required for crystallization changed from a few months to a few weeks, respectively. Initial phases were derived from Single-wavelength Anomalous Diffraction data [3] collected at the selenium absorption edge of a MG491-Nt variant where three isoleucine residues (Ile36, Ile168, Ile205) had been replaced by seleno methionines (see Material and Methods) (Fig 1A). Four selenium sites were located within the crystal asymmetric unit, with two sites related to the other two by a Non-Crystallographic Symmetry (NCS) two-fold axis. Structure determination was then achieved by density modification, averaging between both the less isomorphous crystals (see Materials and Methods) and using the NCS two-fold axis (S1 Fig). The final refined structure, with four subunits in the crystal asymmetric unit (residues 65–204, 67–203, 66–203 and 62–205 for subunits A, B, C and D, respectively; Figs 1B, 2A and 2B), has agreement R_work_ and R_free_ factors of 22.17% and 24.93%, for a seleno methionine MG491-Nt data set at 3.0 Å resolution (Table 1, S2 Fig, PDB entry code 4XNG). The unexpected presence of four subunits in the crystal asymmetric unit had two major implications: i) extensive proteolysis had to have happened in the C-terminal region of the protein (not visible in the determined structures) during crystallization. Four subunits each with 308 residues would give an unacceptably low crystal solvent content of 4%. ii) Four subunits cannot be symmetrically related by the only two-fold symmetry found.

**Table 1:** Data collection, phasing and refinement statistics.

	Full length	Seleno methionine	I3C derivatized
	MG491	MG491-Nt	MG491-Nt
**Data collection**			
Space group	P2_1_2_1_2	P2_1_2_1_2	P2_1_2_1_2
Cell dimensions			
*a*, *b*, *c* (Å)	99.27, 108.73, 66.62	98.26, 108.42, 62.19	97.56, 111.79, 70.98
Wavelength(Å)	0.979	0.979	1.907
Resolution (Å) *	35–3.30 (3.40–3.00)	35–3.00 (3.10–3.00)	35–3.35 (3.45–3.35)
*R*_*pim*_ *	0.024 (0.499)	0.045 (0.345)	0.041 (0.259)
*R*_*meas*_ *	0.044 (0.915)	0.199 (1.554)	0.131 (0.598)
*I* / σ(*I)* *	16.7(2.7)	10.6 (2.4)	19.6 (6.2)
Completeness (%)*	99.3 (93.0)	99.9 (100.0)	99.3 (96.4)
Redundancy *	4.2 (4.1)	19.5 (20.2)	15.1 (7.0)
**Phasing**			
d'' / σ(d'') *		1.15 (0.83)	
Anomalous completeness *		99.2 (94.7)	
Anomalous multiplicity *		10.2 (10.0)	
**Refinement**			
Resolution (Å) *		35–3.00 (3.10–3.00)	
Unique reflections (work)		13851 (694)	
*R*_work_ / *R*_free_		22.17 / 24.93	
No. protein atoms		4588	
No. chains		4	
*B*-factors (Å^2^)			
chain A		42	
chain B		43	
chain C		42	
chain D		40	
R.m.s deviations			
Bond lengths (Å)		0.012	
Bond angles (°)		1.405	
Ramachandran plot			
Disallowed regions (%)		1.99	
Allowed regions (%)		98.1	
Favoured regions (%)		88.5	

*Values in parentheses are for the highest-resolution shell.

### The MG491 structure

The structure determined for MG491 subunits consists of an antiparallel three-helix-bundle, with helices α1 (residues 70–102), α2 (111–145) and α3 (167–203) connected by loops L1 (103–110) and L2 (146–166), respectively (Fig 1A and 1B). Helix α1 is kinked in its central part, due to the insertion of a π-helix turn starting in residue Cys87 (Fig 1B). Only a small and variable number of residues, from three in subunit B to eight in subunit D, could be traced preceding helix α1. Therefore, about sixty residues in the N-terminal region of MG491 appear to be flexible with respect to the subunit three-helix-bundle domain. Differences between the four subunits present an averaged root mean square deviation (r.m.s.d.) for Cα atoms of only 0.32 Å, which increases to 0.70 Å between subunits not related by the NCS two-fold axis, with the largest deviations corresponding to loop L1 and to the central part of loop L2 (residues 152–160).

**Fig 3:**
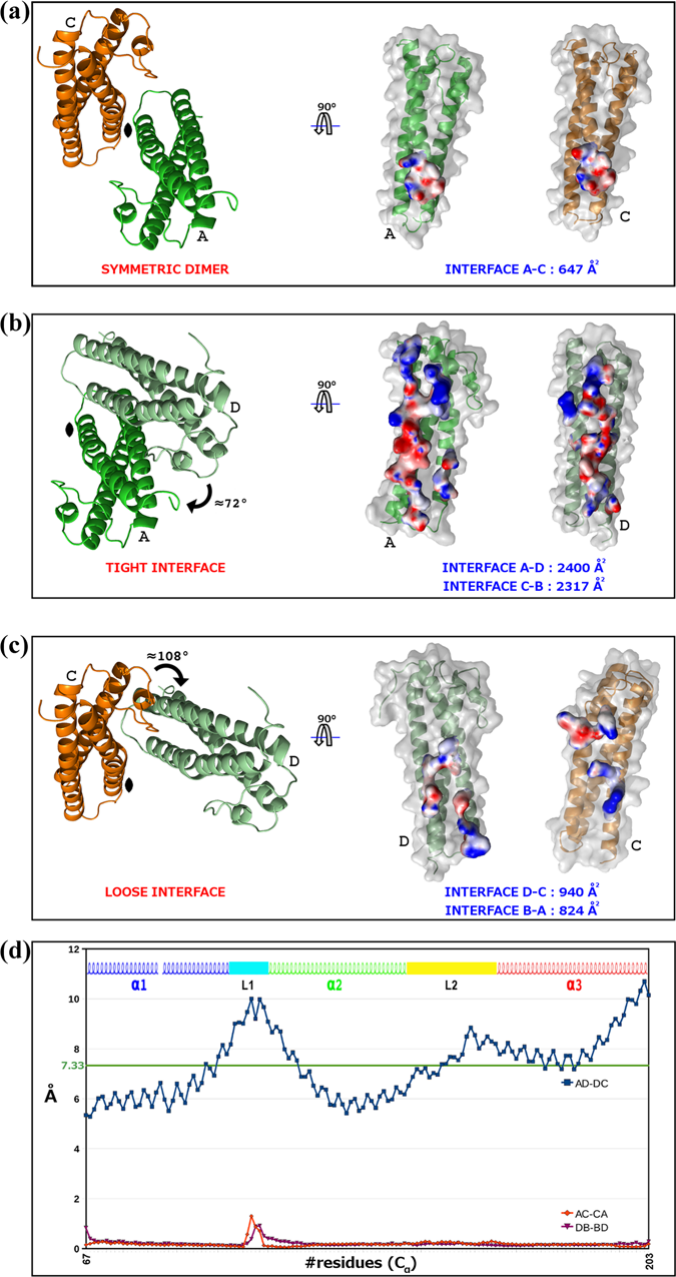
Intersubunit interactions in the MG491 tetramer. (a) The symmetric intersubunit interface, found only between subunits A (in green) and C (in orange), is mainly hydrophobic and involves the C-terminal end of helices α1. (b) The tight interface, found between neighbor subunits related by rotations of ~72° (tight dimers A-D or, analogously, C-B), is the largest intersubunits surface in the MG491 tetramer and is mainly electrostatic. (c) The loose interface, found between neighbor subunits related by rotations of ~108° (loose dimers D-C or, analogously, B-A), is composed of hydrophobic and hydrophilic patches evenly distributed. Red and blue surfaces represent electronegative and electropositive potentials, respectively. (d) Deviations from exact four-fold (blue dotted line) and two-fold (red and magenta dotted lines) molecular symmetries plotted against the residues number. The important asymmetric character of the tetramer is reflected in the large deviations for the four-fold symmetry, with an average value for Cα atoms of 7.33 Å.

**Fig 4:**
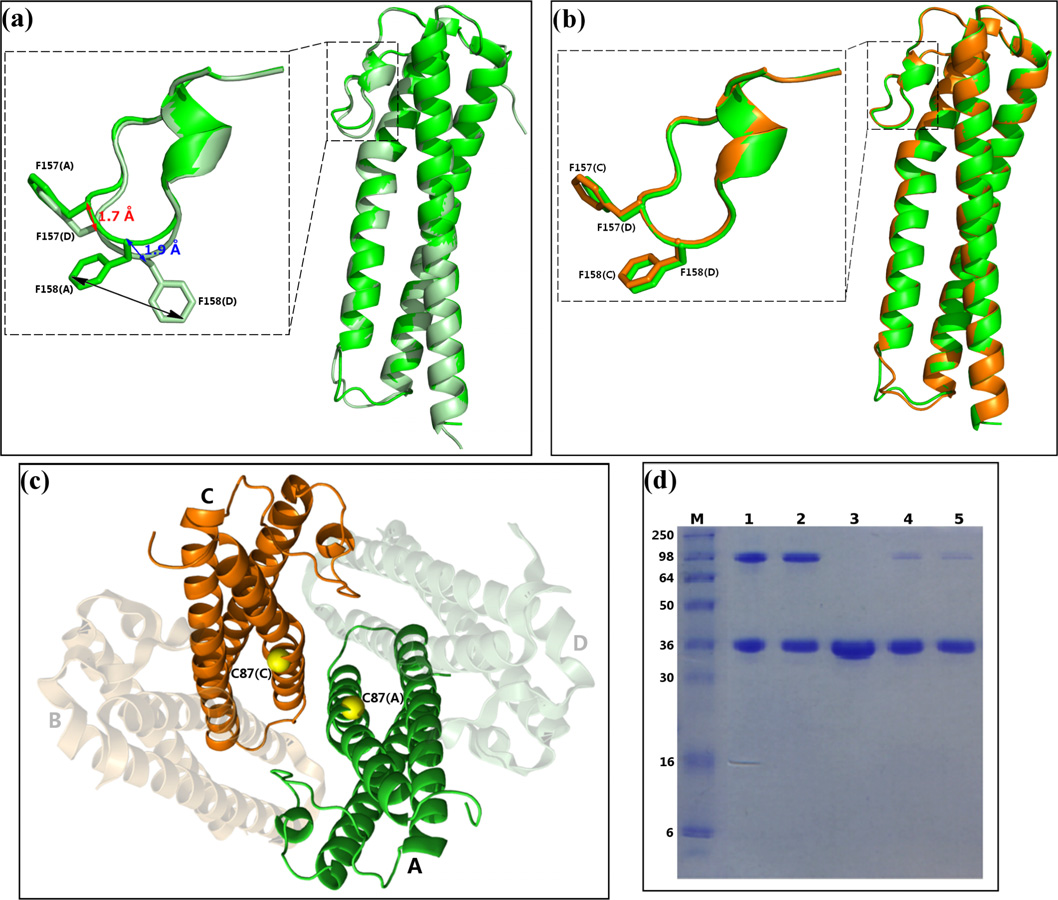
Symmetric and asymmetric features of the MG491 tetramer. Superposition of subunits (a) A and D (displayed in green and pale green, respectively, which are not related by the two-fold molecular symmetry) and (b) A and C (displayed in green and orange, respectively, which are related by the two-fold molecular symmetry). Superpositions of loops L2 are presented in some detail in the inserts, with the side chains of Phe157 and Phe158 explicitly shown. (c) In the MG491 tetramer Cys87 residues (yellow spheres) are close to each other for subunits A and C (green and orange, respectively) remaining far away for subunits B and D (shadow representation). (d) SDS-PAGE gel analysis of the formation of disulfide bonds in MG491. Lane M corresponds to molecular weight markers. MG491 samples treated with the oxidizing agent DMSO and not heated (lane 1), treated with DMSO but heated for 10 minutes at 80°C (lane 2), not treated with DMSO (lane 3), treated with DMSO and then loaded into the gel in the presence of the reducing agent DTT (lane 4) and same as lane 4 but heated for 10 minutes at 80°C (lane 5). Formation of disulfide bridges, for about 50% of subunits, is clear in lanes 1 and 2.

The four MG491 subunits found in the crystal asymmetric unit present three different types of intersubunit interfaces that were named symmetric, tight and loose (Figs 2B and 3A–3C). The symmetric interface, with a total buried area of ~650 Å^2^, corresponds to interactions across the two-fold symmetry axis and involves only subunits A and C, while subunits B and D do not contact with each other (Fig 3A). The tight interfaces, with a total buried area of ~2*1200 Å^2^, correspond to the two interfaces between subunits in the pairs A/D and C/B (within each pair subunits are related by ~72° rotation) (Fig 3B). The loose interfaces, with a total buried area of ~2*450 Å^2^, correspond to the two interfaces between subunits in the pairs A/B and C/D (within each pair subunits are related by ~108° rotation) (Fig 3C). Therefore, the four subunits found in the crystal asymmetric unit present a network of (extensive) intersubunit interactions strongly suggesting that the MG491 molecule can form tetramers, in agreement with studies by gel filtration, crosslinking with glutaraldehyde and nano-ElectroSpray Ionization Mass Spectrometry (S3 Fig). Despite the fact that the four subunits in the tetramer are structurally similar, as reflected by the low r.m.s.d. values, they are placed in two different environments. Therefore, two types of subunits can be identified according to the residues that participate in the tight and loose interfaces of each subunit. The organization of the MG491 tetramer, with only a two-fold symmetry, can be defined as a dimer (C2 molecular symmetry) of asymmetric pairs of subunits. Quantification of the deviation from an accurate four-fold molecular symmetry gives an average for the Cα atoms of all the residues of 7.3 Å (Fig 3D). Attempts to form a regular (symmetric) oligomer using only the interactions corresponding to the tight interface would result in a helical aggregate with four subunits at most due to steric clashes (S4A Fig). In turn, oligomerization using only the loose interface would result in helical aggregates with three subunits at most (S4B Fig). Loop L2 mediates interactions in both the tight and the loose interfaces presenting a different conformation in each interface. In the loose interface the backbone in the central part of loop L2 moves towards the neighbor subunit by ~2 Å, with the side chain of Phe158 flipping to a different conformation about 7.5 Å away (Fig 4A and 4B). Interestingly, the conformational changes observed for loop L2 result in similar intersubunit interactions for the two interfaces when analyzed with LigPlot^+^ [28]. In particular, interaction of Phe157 with Gly91 in the tight interface is mirrored as interaction of Phe157 with Gly80 in the loose interface (S5 Fig).

In the MG491 tetramer, the four Cys87 residues (Cys87 is the only cysteine in the whole MG491 sequence) are located close to each other (Fig 4C), in particular across the symmetric interface, suggesting the possible formation of disulfide bonds between subunits. However, in the structure determined disulfide bonds are absent, which might be due to the presence of reducing agents required for crystallization. Following an established oxidation protocol to form disulfide bonds *in vitro* [29], the protein was diluted in 1x PBS (pH 7.5) to a final concentration of 1 mg/ml and incubated for 2–4 h in 1% (v/v) DMSO, resulting in the formation of intersubunit disulfide bridges but only between ~50% of the subunits (Fig 4C). This result supports a departure of symmetry in the molecular organization of MG491 that would agree with disulfide bridges being formed only between the two subunits at the symmetric interface of the MG491 tetramer.

The sequence alignment (http://espript.ibcp.fr) [30] between MG491 and its *M*. *pneumoniae* homolog, P41, gives an overall identity of 53% mainly due to the high identity found for the proteins N-terminal regions (until about residue 203 in MG491, Fig 1A). In particular, the most conserved regions are for MG491 helices α1 and α3 as well as for loops L1 and L2. Accordingly, the MG491 structure is expected to be well preserved in the *M*. *pneumoniae* protein P41.

### Characterization of MG491 mutant strains

**Fig 5:**
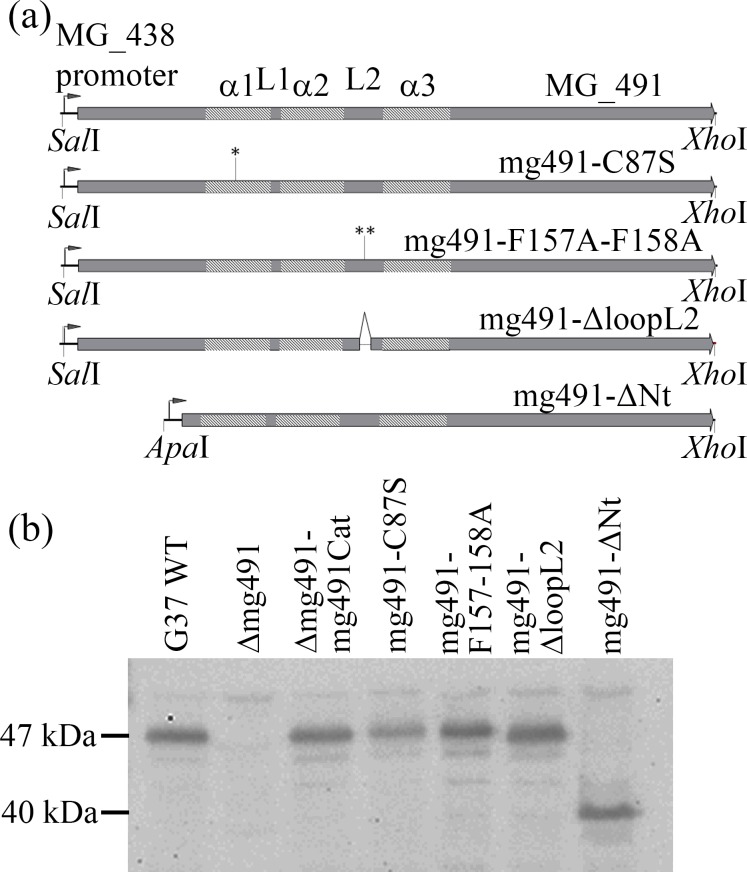
Engineering of the *M*. *genitalium* MG491 mutant strains. (a) Schematic representation of the different MG491 alleles under the control of the MG438 promoter. These alleles were introduced in a plasmid containing a mini-transposon using the indicated restriction sites and electroporated into Δmg491 cells. Coding sequences for helices α1, α2 and α3 and loops L1 and L2 are indicated. The Cys87 to serine mutation is labeled with an asterisk (*) in the MG_491C87S allele. A double asterisk (**) is pointing to the F157A and F158A mutations in the MG491-F157A-F158A allele. Deletion of the 463–480 bp region from MG_491 coding for residues 155 to 160 is labeled with lines in the MG491-loopL2 allele. (b) Western blot analysis of G37 wild type cells, Δmg491 cells and cells from transformant colonies containing the alleles in (a) using a heterologous polyclonal serum anti-P41 from *M*. *pneumoniae*. Expression of MG491 is detected in wild type G37 cells and in the transformant cells except for the Δmg491 strain. A lower amount of MG491 was observed in the Δmg491-C87S strain and a band with an approximate molecular weight of 40 kDa was detected in the mg491-ΔNt strain.

To study the biological relevance of the unique molecular organization of MG491 and to further investigate the function of this protein, three structure-guided *M*. *genitalium* mutant strains were engineered with mutations in residues directly involved in the interactions between subunits. These three protein variants were: i) Cys87 replaced by a serine; ii) Phe157 and Phe158 substituted both by alanines and iii) the peptide from Asn155 to Lys160, corresponding to a large fragment of loop L2, deleted (Fig 1A). An additional mutant strain lacking the N-terminal region of MG491 (residues 1–61) was also engineered to gain insight into the function of this region. Mutant alleles mg491C87S, mg491F157A-F158A, mg491ΔloopL2 and mg491ΔNt were introduced in pMTn*cat* plasmids [23] under the control of the MG438 promoter (Fig 5A). These mini-transposons were electroporated into cells from *M*. *genitalium* Δmg491 null mutant strain lacking MG_491 [20]. One colony from each transformation experiment was selected for the different alleles and named mg491-C87S, mg491-F157A-F158A, mg491-ΔloopL2 and mg491-ΔNt, respectively. Transposon insertion sites were investigated by direct genome sequencing and all the selected transformants showed transposon insertion sites in genes other than those involved in the terminal organelle architecture and/or gliding motility functioning (Table 2).

**Table 2:** Transposon insertion points.

Strain	Insertion point	Strand	Gene	Function	Disruption^a^
Δmg491-mg491cat	468883	-	MG_370	Pseudouridine synthase	D
Mg491-C87S	517561	+	MG_414	Conserved hypothetical protein	D
mg491-F157A-F158A	37056	-	MG_032	Conserved hypothetical protein	D
mg491-ΔloopL2	564886	-	MG_460	L-lactate dehydrogenase	ND
mg491-ΔNt	517115	+	MG_414	Conserved hypothetical protein	D

^a^ D, disruptive insertion; ND, non-disruptive insertion.

**Fig 6:**
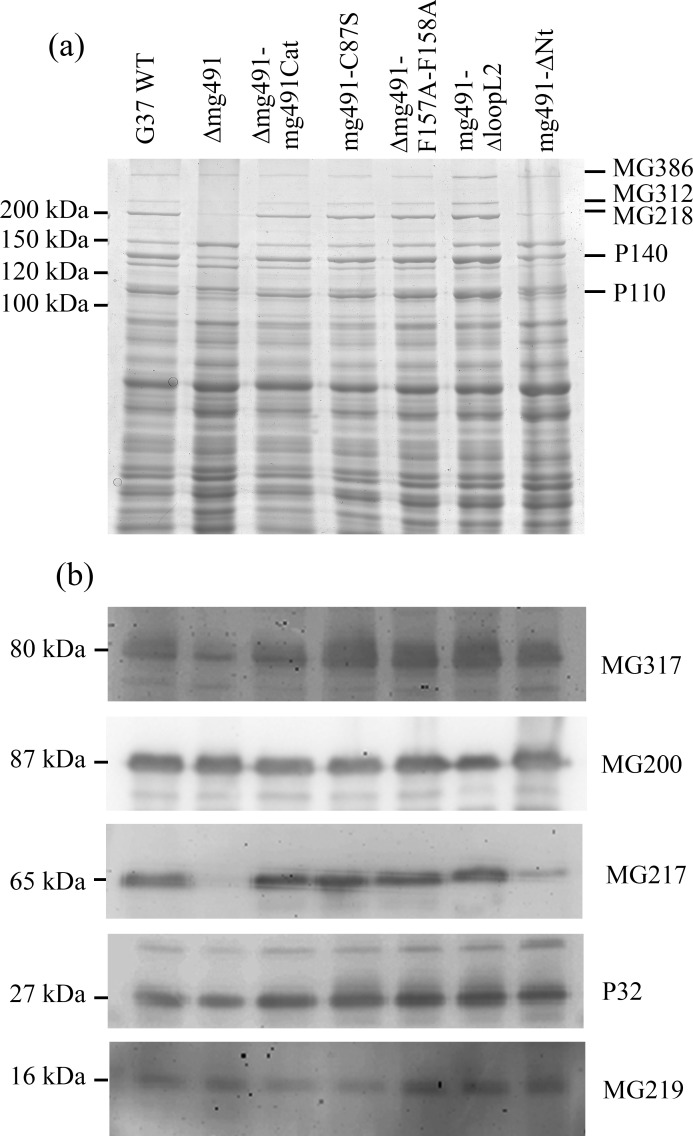
Protein profile and Western immunoblot analysis of the *M*. *genitalium* MG491 mutant strains. (a) SDS-PAGE analysis of G37 wild type and mutant strains Δmg491, Δmg491-mg491cat, Δmg491-mg491-C87S, Δmg491-mg491-F157A-F158A, Δmg491-mg491-loopL2 and Δmg491-mg491ΔNt. Bands for proteins MG386, MG312, MG218, P140 and P110 were identified by their typical electrophoretic mobility [18]. The levels found for the cytadherence accessory proteins MG312, MG218 and for the P140 and P110 adhesins are highly reduced in the Δmg491 strain. Normal levels of these proteins are recovered in strains Δmg491-mg491cat, Δmg491-mg491-C87S, Δmg491-mg491-F157A-F158A and in Δmg491-mg491-loopL2, but not in Δmg491-mg491ΔNt. (b) Levels of the cytadherence accessory proteins MG317, MG200, MG217, P32 and MG219 as observed by western immunoblot analysis of the *M*. *genitalium* mutant strains produced in this study. Lanes are the same as described for panel (a). The levels of MG317, MG217 and P32 are reduced in the Δmg491 and Δmg491-mg491ΔNt mutant strains but these proteins are found at normal levels in the Δmg491-mg491cat, Δmg491-mg491-C87S, Δmg491-mg491-F157A-F158A and Δmg491-mg491-loopL2 mutant strains.

Upon introduction of the wild type allele in Δmg491 cells, steady-state levels of the MG491 protein were restored in the Δmg491-mg491cat strain (Fig 5B). Normal levels of MG491 were also observed in the mg491-F157A-F158A, mg491-ΔloopL2 and mg491-ΔNt mutant strains (Fig 5B). However, a lower amount of MG491-Cys87Ser was detected in Δmg491-C87S cells, suggesting that Cys87 might play an important role in protein stability. The apparent molecular weight of the deletion variant protein MG491ΔNt was ~40 kDa, in agreement with the expected value. Lower levels of MG491 have already been shown to correlate well with the existence of several downstream events in terminal organelle related proteins [20]. Therefore, it was not surprising to observe in Δmg491 cells, a drastic decrease in the amount of adhesion proteins P110 and P140, and of most of the cytadherence accessory proteins (Fig 6A and 6B). However, these cells exhibited normal amounts of the cytadherence accessory proteins MG200 and MG219. The adhesin and cytadherence accessory proteins levels were also restored upon reintroduction of the wild type *MG_491* allele in the *M*. *genitalium* Δmg491 strain and a similar effect was observed in the transformants containing the mutant alleles mg491-C87S, mg491-F157A-F158A and mg491-ΔloopL2. In contrast, the levels of adhesins and cytadherence accessory proteins were not restored after the introduction of the mutant allele in mg491-ΔNt, indicating that the N-terminal region of MG491 has an important role in the formation and stabilization of the terminal organelle (Fig 6A and 6B).

### Cell morphology and motile properties of the MG491 mutant strains

**Fig 7:**
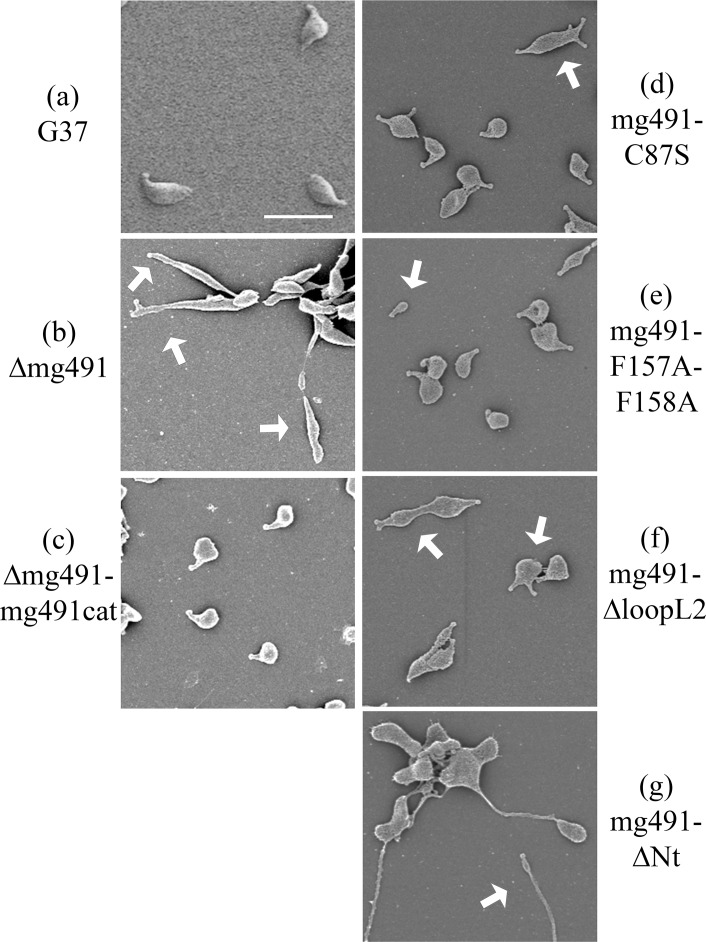
Scanning electron micrographs of *M*. *genitalium* G37 wild type and MG491 mutant strains. (a) *M*. *genitalium* G37 cells showing the typical flask-shaped morphology conferred by the presence of a terminal organelle. (b) Δmg491 cells showing filamentous morphologies pointed by white arrows. (c) Δmg491-mg491cat cells showing normal terminal organelles and flask-shaped morphologies. (d) mg491-C87S cells showing also flask-shaped morphologies, but many of them exhibiting multiple terminal organelles (white arrow). (e) mg491-F157A-F158A cells also showing flask-shaped morphologies but exhibiting minute cells (<0.35 μm) pointed by a white arrow. (f) mg491-ΔloopL2 cells showing the flask-shaped morphology but exhibiting multiple terminal organelles (white arrow). (g) mg491-ΔNt cells showing a filamentous cell morphologies (white arrow). Bar is 1 μm.

**Fig 8:**
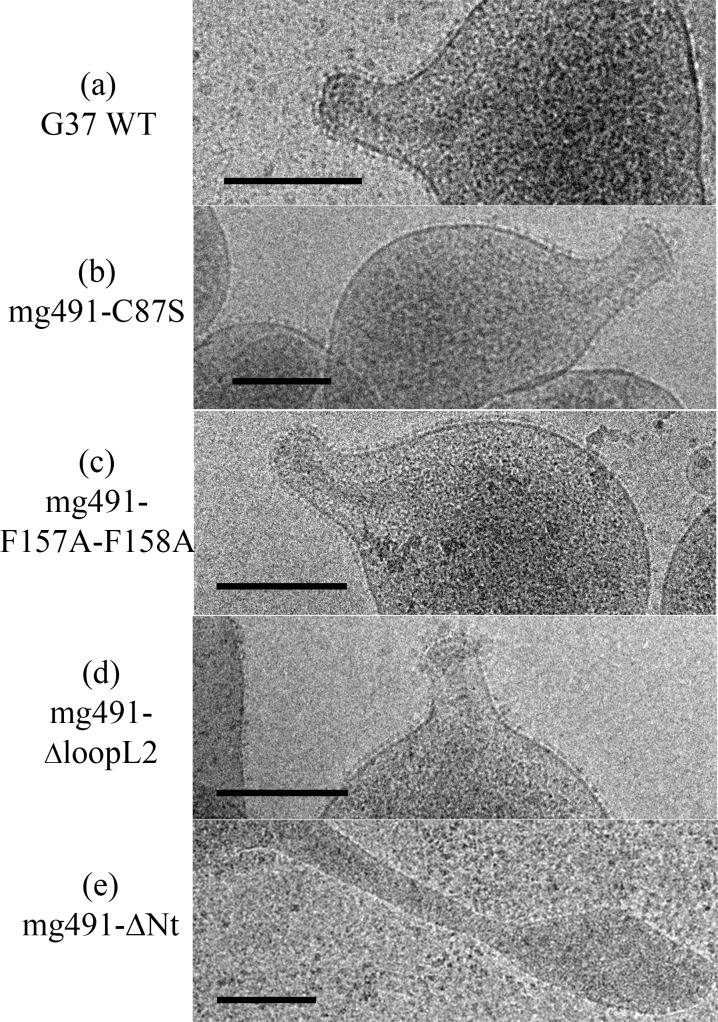
Cryo-electron microscopy analysis of *M*. *genitalium* G37 wild type and MG491 mutant strains. (a) G37 wild type cells showing the cytoskeleton supporting the terminal organelle and the nap layer surrounding the cell membrane. (b) mg491-C87S, (c) mg491-F157A-F158A and (d) mg491-ΔloopL2 cells, exhibiting normal cytoskeletons and nap layers. (e) mg491-ΔNt cells showing no internal terminal organelle structure inside the filament. Bars are 0.2 μm.

Cells from the Δmg491 strain showed a filamentous morphology when observed by scanning electron microscopy (Fig 7A). The characteristic flask-shaped morphology typically observed in wild type cells was restored in the Δmg491-mg491cat strain (Fig 7B). The gliding properties were also restored in these cells, showing no significant differences when compared to those exhibited by G37 wild type cells (Table 3, S1 and S2 Movies, S6 Fig). Cells from the mg491-C87S strain showed normal terminal organelles (Fig 7C) but this strain also presented a high frequency of cells bearing multiple terminal organelles, which correlated with a reduced number of motile cells and a slower mean velocity as measured by time lapse microcinematography (Table 3, S3 Movie). When examining microcinematographies of G37 wild type cells, 18% of the motile cells show one or more resting periods. These resting periods are short and seem not to be related to cell division. Remarkably, the frequency of motile cells showing resting periods in mg491-C87S strain was 49%, indicating that the high frequency of non-motile cells might be a consequence of these resting periods. Likewise, a large amount of cells bearing multiple terminal organelles was also observed when examining the mg491-ΔloopL2 strain (Fig 7E) but these cells showed, in addition, a drastic decrease in different gliding motility parameters (Table 3, S4 Movie) and a low hemadsorption activity (S6 Fig). Moreover, both strains showed normal levels of all known proteins involved in gliding motility (Fig 6A and 6B) and exhibited no significant changes in the overall terminal organelle architecture (Fig 8). These data suggest that gliding motility impairments detected in these strains are a direct consequence of the mutations introduced in MG_491. In contrast, the gliding properties and the frequency and architecture of terminal organelles in mg491-F157A-F158A cells were similar to those of wild type cells (S5 Movie and Figs 7D and 8C). However, this variant shows a lower hemadsorption activity than G37 wild type (Table 3, S6 Fig) and a large amount of minute cells smaller than 0.35 μm in size as revealed by electron microscopy (Fig 7D and Table 3). Cells from this strain were stained with Hoechst 33342, examined by time lapse microcinematography and finally visualized by epifluorescence microscopy. Most of the minute cells analyzed (93.3%) showed no detectable fluorescence after staining with Hoechst indicating that these cells did not contain detectable amounts of DNA. Among these non-fluorescent cells, 53 of them (54.1%) were found motile during the examination period (S7 Fig), indicating that these minute cells were consequence of terminal organelle detachments. Such minute cells are rarely observed in *M*. *genitalium* G37 wild type strain. In contrast, cell detachments are frequently observed when the terminal organelle is not properly anchored to the cell body. Minute cells were previously described to be the result of terminal organelle detachments from the main cell body in *M*. *genitalium* cells lacking the C-terminal region of MG491 [22] and also in *M*. *pneumoniae* cells with a disrupted MPN311 gene, which codes for the P41 protein (S1 Table) [30]. Thus, the presence of minute cells in the mg491-F157A-F158A strain suggests that the intersubunits interactions promoted by Phe157 and Phe158 are required for the proper assembly of MG491, possibly playing an important role in the stabilization of the protein quaternary structure. However, oligomerization of protein variant Phe157Ala-Phe158Ala appears similar to the wild type protein presenting, surprisingly, even a slightly increased stability (S8 Fig). Finally, electron microscopy analysis of the mg491-ΔNt strain revealed the presence of a large amount of cells with filamentous morphology and the absence of rods inside these filaments (Fig 8E), similar to what was observed when examining the parental Δmg491 strain [20]. Moreover, no motile cells were observed for this strain (S6 Movie), suggesting that MG491 is involved in the assembly of the terminal organelle and in its stabilization through the protein N-terminal region.

**Table 3:** Gliding parameters, cell morphology and hemadsorption of *M*. *genitalium* G37 wild type and MG_491 mutant strains.

Strain	Motile cells (%)	Mean velocity (μm/s)±SE^b^	Resting Periods (%)	Multiple Terminal Organelles (%)	cells <0.35 μm (%)	K_D_ ^a^ (RBC·μL^-1^) ± SE^b^
G37	85.0	0.121 ± 0.006	18	4.9	1.0	344.47 ± 82.18
Δmg491-mg491cat	82.3	0.125 ± 0.003	19	5.5	1.5	238.96 ± 47.71
mg491-C87S	19.0*	0.098 ± 0.005*	49*	12.5*	2.5	390.32 ± 53.39
mg491-F157A-F158A	80.7	0.100 ± 0.005*	24	5.6	13.6*	3645.5 ± 1662.7*
mg491-loopL2	29.6*	0.033 ± 0.004*	29	26.2*	1.0	6482.1 ± 1424.5*

* Significant difference with the *M*. *genitalium* G37 wild type (p<0.05).

^a^ K_D,_ dissociation constant between the mycoplasma cells and the red blood cells.

^b^ SE, standard error

## Discussion

Human pathogen *M*. *genitalium*, from the *pneumoniae* cluster of mycoplasmas, presents a flask-shaped morphology conferred by a polar structure, known as terminal organelle, which neither structurally nor functionally is yet well understood. MG491 protein from *M*. *genitalium* shares a high sequence identity with the *M*. *pneumoniae* protein P41, which is known to be an important component of the terminal organelle in *M*. *pneumoniae* and has been located at the base of the electron-dense core [31]. The location of MG491 in the terminal organelle of *M*. *genitalium* was also supported by the finding that a 25-residue region interacts specifically with MG200 [25], a protein that had been shown to be involved in gliding motility [23,24]. The structural characterization of MG491 in this work, indicates that MG491 subunits are composed of three distinct regions with a 60-residue N-terminus, a central three-helix-bundle spanning about 150 residues and a C-terminal region that contains the residues that interact with MG200 and appears to be mostly unstructured (Fig 1A). Only the central helix-bundle is well defined in the electron density maps of crystals from several constructs of MG491 (Fig 1B). All the solved crystal structures contain four crystallographically independent subunits, which are interwoven by a network of interactions with each other (Fig 2A and 2B). Surprisingly, each one of this tetrameric ensembles is organized with only one two-fold symmetry axis that relates pairs of subunits (Fig 3A). These pairs can be defined in two alternative ways referred as loose or tight according to the extension of the interacting interface between the two subunits in the pair (Fig 3B and 3C). Steric clashes make it impossible to model regular oligomers containing only one kind of these interacting surfaces (S4 Fig). The biological relevance of this unique organization has been assessed by the characterization of *M*. *genitalium* mutant strains with alterations in residues involved in intersubunit interactions. The MG491 variant Cys87Ser preserves normal levels of all the other terminal organelle proteins but presents a very significant reduction in motility (Table 3), comparable to the effects observed in deletion mutants of whole proteins involved in gliding motility [24,32]. Terminal organelle development is synchronized with cell division and cytokinesis appears to be highly coordinated with gliding motility, which is also essential for segregation of the terminal organelles to the opposite cell poles. In this way, alterations in motility often result in the presence of cells bearing multiple terminal organelles [32–34]. In contrast, cells from mg491-C87S mutant strain show only a very modest increase (7.6%) in the frequency of cells with multiple terminal organelles. Moreover, the gliding velocity of these cells is not significantly lower than that exhibited by wild type cells (Table 3). The large number of non-motile cells in the mg491-C87S mutant is strongly correlated with an increased frequency of cells showing resting periods, rather than with the presence of cells stalled in the cytokinesis process, as observed in other gliding mutants [24,32]. The higher frequency of resting periods in the mg491-C87S mutant strain suggests that the Cys87 residue of MG491 might have an important role in the regulation of gliding motility. Interestingly, the frequency of resting periods was also found increased in *M*. *genitalium* cells lacking the EAGR box from MG200 [16], reinforcing the relevance of the interplay between MG200 and MG491 in the regulation of gliding motility [22]. The strikingly severe effects of a single point mutation on the only cysteine residue suggests a major role for this cysteine that is likely related with the asymmetric formation of intersubunit disulfide bridges observed *in vitro* (Fig 4D). Only the two subunits in the tetramer of MG491 that interact across the molecular two-fold symmetry axis (subunits A and C in Figs 3 and 4) are expected to participate in this interaction, while the cysteine residues of the other two subunits would remain reduced or available for different interactions. MG491 variants designed to alter the tight and loose interfaces by deleting the central part of loop L2 (ΔloopL2) or replacing two of the loop residues (Phe157Ala-Phe158Ala) also resulted in significant changes in cell motility and cell morphology (Table 3 and Figs 7E and 7F and 8C and 8D). As expected, alterations in the deletion variant ΔloopL2, which also includes residues Phe157 and Phe158, are stronger than those observed for the Phe157Ala-Phe158Ala variant and, accordingly, the frequency of cells with multiple terminal organelles is higher in ΔloopL2 cells (Table 3). In contrast, the MG491 double mutation variant Phe157Ala-Phe158Ala showed a significant increase in the amount of minute cells or terminal organelles detached from the main cell body despite the fact that no clear changes were observed *in vitro* for the oligomerization of the variant (S8 Fig). The increased frequency of minute cells strongly supports that Phe157 and Phe158 residues have a main role in the stability of the wheel complex or in the interactions of the wheel complex with the rod.

Despite the complexity of the terminal organelle of mycoplasmas, here we show that this structure can be a reachable target for a thorough characterization zooming out in resolution from atomic to cellular levels. In this work, the structural information obtained from the crystal structure of MG491 has guided the preparation of several *M*. *genitalium* mutant strains of this protein. As a result, the motility and morphology of *M*. *genitalium* cells have been importantly affected, providing, for the first time, information on how the structure of a protein relates with the organization, stabilization and functioning of the terminal organelle. Motile mycoplasmas with spreading deficiencies are associated to a reduced infectivity [17,35], which emphasizes the relevance of MG491 in the virulence of *M*. *genitalium*.

## Materials and Methods

### Bacterial strains and growth conditions

The *E*. *coli* XL1-Blue strain was used to amplify the plasmids used in this study and was grown on LB agar plates or liquid LB media overnight. Ampicillin was added at 0.1 mg/ml. *M*. *genitalium* G37 wild type and mutant strains were grown in SP-4 broth at 37°C under 5% (v/v) CO_2_ in tissue culture flasks (from TPP, Switzerland) until mid-log phase of growth. Transformant colonies were isolated on SP-4 agar plates supplemented with 2 μg/ml tetracyclin and 34 μg/ml chloramphenicol.

### Cloning, expression and purification of MG491 and some variants

The coding sequence of the MG_491 gene was amplified from *M*. *genitalium* G37 wild type genomic DNA with oligonucleotides 5MG491 and 3MG491 and ligated into a pBE plasmid [36]. The triplet coding for Trp232 from the MG491 protein was changed from TGA to TGG by amplification of this plasmid with oligonucleotides MutMG491PA and MutMG491PB and circularization of the amplicon with T4 DNA ligase. Afterwards, the sequence coding for this mutated version of the full length MG491 protein was cloned between *Nde*I and *Xho*I restriction sites of a pET21d expression vector (Novagen, Madison, WI, USA), which also codes for a C-terminus hexa-histidine tag. The resulting vector was transformed into *E*. *coli* BL21 (DE3) cells and the transformant cells were plated on LB/agar plates supplemented with ampicillin. After checking the correctness of the DNA sequence, the transformant cells were cultivated in 1 l LB medium containing 0.1 mg/ml ampicillin and induced overnight with 1 mM IPTG at 20°C with constant shaking after reaching an OD_600_ of ~0.6. Subsequently, the cells were harvested by centrifugation at 4500 x*g* for 15 min at 4°C. The pellet was resuspended in lysis buffer (0.02 M Tris-HCl (pH 8.0), 0.5 M NaCl, 0.02 M imidazole, complete EDTA free protease inhibitor (Roche Diagnostics, Mannheim, Germany)) and the cells disrupted by sonication. The total lysate was then centrifuged twice for 20 min at 45000 x*g* to remove cells debris and filtered through a 0.22 μm filter. The his-tagged MG_491 gene product present in the resulting supernatant was firstly purified through a 5 ml HisTrap HP column (GE Healthcare Life Sciences, Uppsala, Sweden) previously equilibrated in 0.05 M Tris-HCl (pH 8.0) buffer containing 0.5 M NaCl and 0.02 M imidazole, concentrated to a suitable volume and then loaded on a Superdex 200 16/60 gel filtration column (GE Healthcare Life Sciences, Uppsala, Sweden) equilibrated in 0.05 M Tris-HCl (pH 8.0) containing 0.15 M NaCl.

To obtain the phases for the X-ray structure determination several methionine residues (absent in the MG491 sequence) were introduced based on secondary structure element predictions, in positions corresponding to Ile36, Ile168, Ile205 and Ile313. A new expression vector was prepared (pET21d-MG491-B) using pET21d-MG491 as template and the oligonucleotide primers containing the appropriate target substitutions (see S1 Table). Limited proteolysis experiments performed with Trypsin on a MG491 sample generated a fragment of about 30–35 kDa with an intact N-terminal (revealed by Edman sequencing), which suggested that the C-terminal region is more accessible and thus more susceptible to proteolysis. Given this, and using the pET21dMG491-B vector as template and the appropriate primers (see S1 Table), a shorter variant of the protein was designed spanning MG491 residues 1 to 308 (MG491Δ308). The resulting PCR fragment was finally cloned into a pOPINE expression vector [37], which encodes for an extra lysine and a hexa-histidine tag at the C-terminal end of the construct. This new vector was then transformed into *E*. *coli* BL21 (DE3) cells and the MG491-Nt protein was expressed and purified following the same protocol used to prepare the full length protein. Additionally, the seleno methionine-labeled MG491-Nt protein was produced by growing a 0.1 l pre-culture overnight at 37°C in presence of 400 μl L-methionine at 10 mg/ml, 2 ml of 50% (w/v) glucose (freshly prepared and filtered through a 0.22 μm filter) and the appropriate antibiotic. Cells were then recovered by centrifugation at 4500 x*g* for 15 min, washed three times with 1x PBS, to remove the L-methionine that has not been incorporated by the cells, and finally resuspended in 2 ml 1x PBS. This cell pellet was then used to inoculate 1 l of SelenoMet media (Molecular Dimensions Ltd., Newmarket, UK) in presence of 9 ml L-seleno methionine at 10 mg/ml and supplemented with OnEx solutions 1, 2 and 3 from the Overnight Express Autoinduction Systems 1 (Novagen, Madison, WI, USA). Cells were grown for 6 h at 37°C, then the temperature was lowered to 25°C and growth was continued for 20 h with constant shaking before harvesting. The seleno methionine-labeled MG491-Nt protein was finally purified following the same protocol used for the full length MG491 and MG491-Nt proteins. Under these conditions, the proteins eluted as single peaks consistent with tetramers of ~200 kDa, respectively. The propensity of MG491 to form tetramers was also assessed and confirmed by crosslinking with glutaraldehyde [38] and by nano-ElectroSpray Ionization Mass Spectrometry (S3 Fig).

### Crystallization and heavy atom derivative preparation

Crystals of the full length MG491, MG491-Nt and seleno methionine-labeled MG491-Nt (respectively at concentrations of 10 mg/ml, 8 mg/ml and 15 mg/ml), were grown at 20°C by the vapour-diffusion method over a reservoir containing 0.2 M lithium sulphate monohydrate, 25% (w/v) PEG 3350 and 0.1 M Bis-Tris (pH 6.5) or 0.1 M HEPES (pH 7.5) or 0.1 M Tris-HCl (pH 8.5). Before data collection crystals were transferred to a drop of reservoir solution containing 15% (v/v) propylene glycol as cryoprotectant and flash-cooled in liquid nitrogen. Crystals of MG491-Nt, soaked for 10 to 60 sec in a drop of mother liquor containing 12.5–100 mM of 5-amino-2,4,6-triiodoisophthalic acid (I3C, Sigma), were then rapidly back-soaked [39] in a drop of mother liquor containing 15% (v/v) propylene glycol as cryoprotectant and flash-cooled in liquid nitrogen.

### Data collection and structure determination

X-ray diffraction data was collected at 100 K on beamlines ID23-1 [40] and ID29 [41] (ESRF, Grenoble, France) for crystals of the full length MG491 and seleno methionine-labeled MG491-Nt proteins and on beamline PROXIMA1 (SOLEIL, Gif-sur-Yvette, France) for crystals derivatized with the I3C compound. All beamlines used were equipped with PILATUS 6M-F detectors [42]. For an optimal measurement of the anomalous differences on the seleno methionine-labeled MG491-Nt crystals, a MiniKappa goniometer mounted on beamline ID29 (ESRF, Grenoble, France) was used to re-orient the investigated crystal before data collection, aligning a crystallographic axis along the rotation axis such that Bijvoet mates were on the same image [43]. Data were integrated with XDS [44,45], the output unmerged XDS ASCII file reflection.HKL was then converted to MTZ format by COMBAT and a list of free reflections generated (CCP4 Program Suite v6.4.0). The resulting reflection files were finally scaled with SCALA [46,47]. Phasing statistics for each data set containing anomalous differences were assessed with the processing software XDS, SCALA, XPREP (Bruker AXS Inc., Madison, Wisconsin, USA.) or SHELXC from the SHELX suite [48,49]. All crystals from the different protein constructs belonged to the orthorhombic space group P2_1_2_1_2, with unit cell parameters in the range of a = 96–98 Å, b = 107–112 Å and c = 62–70 Å, indicating an important non-isomorphism not only between native and derivative crystals but also between different derivative crystals (Table 1 and S1 Fig). The HKL2Map GUI interface [50] was used to run the SHELX triad. Initial maps, obtained from the seleno methionine-labeled MG491-Nt data set with the highest anomalous signal, were improved by extensive density modification procedures including averaging between the less isomorphous crystals with programs DM and DMMULTI [51,52]. The command-line utility *phenix*.*get_cc_mtz_mtz*, from Phenix suite [53], which uses RESOLVE [54], was used to facilitate comparisons between density maps with origin shifts compatible with the space group symmetry. The model was completed and refined in rounds of manual rebuilding and restrained refinement with REFMAC [55], using TLS and isotropic B-factors only in the final stages of refinement. The quality of the final model was validated using MolProbity [56] and PROCHECK [57] (Table 1). Interacting surfaces were analyzed with Pymol (The PyMOL Molecular Graphics System, Version 1.5.0.4 Schrödinger, LLC) and the electrostatic representation was generated with the APBS plug-in.

### Quantification of the deviation from perfect C2 and C4 molecular symmetry

Deviations from perfect C2 and C4 cyclic symmetry were calculated for the Cα atoms as the interatomic distances differences (null when the symmetry is perfect) between pairs of subunits [58].

### Isolation of *M*. *genitalium* MG491 mutant strains

The pMTnMG491cat plasmid containing a mini-transposon bearing the coding sequence of MG_491 under the control of the MG438 promoter was amplified using the phosphorylated oligonucleotide P-C87SMG491/5 and the oligonucleotide C87SMG491/3. The resulting PCR fragment was circularized by ligation of the blunt ends to obtain the pMTnMG491C87S plasmid. Similarly, pMTnMG491cat plasmid was amplified using the oligonucleotide FFAAMG491/5 and the phosphorylated oligonucleotide P-loop/3. The amplicon was circularized by ligation to obtain the pMTnMG491FA plasmid. The pMTnMG491cat plasmid was also amplified using the oligonucleotide loop/5 and the phosphorylated oligonucleotide loop/3. The PCR product was circularized by ligation to obtain pMTnMG491loop plasmid. Finally, the 855 bp 3’ coding sequence of MG_491 was amplified using oligonucleotides MG491pr438ct/5 and MG491/3. The PCR fragment was excised with *Apa*I and *Xho*I restriction enzymes and ligated into a pMTncat plasmid [23] to obtain the pMTnMG491ΔNt plasmid. The four constructed plasmids were electroporated into Δmg491 cells and the transformants were isolated in SP-4 agar plates supplemented with tetracyclin and chloramphenicol. Transposon insertions were considered to disrupt a gene sequence when they fell within the 5'-most 80% of the ORF and were located after at least three codons from the start of the protein-coding region [3].

### SDS-PAGE and western immunoblotting

Total protein extracts of mycoplasma strains were electrophoresed in standard SDS-PAGE gels and stained with Coomassie Brilliant Blue or transferred electrophoretically to PVDF membranes following standard procedures [59]. PVDF membranes were probed with anti-MG217 at 1:500 dilution [60], anti-HMW3 at 1:5 000 dilution [61], anti-P41 at 1:1 000 dilution [62], anti-P32 at 1:2 000 dilution, anti-MG200 at 1:5 000 dilution [24] and anti-MG219 at 1:1 000 dilution.

### Quantitative hemadsorption assay

The hemadsorption activity of *M*. *genitalium* G37 wild type and MG491 mutant strains were quantitatively determined by flow cytometry as previously described [63] using a FACSCalibur (Becton Dickinson). The fraction of non-attached mycoplasma cells was plotted *vs* the concentration of red blood cells and fitted to inverse Langmuir Isotherm curves by iteration using the KaleidaGraph software (Synergy). The Wald test was used to find statistically significant differences in the dissociation constant (K_D_) of the different strains with the G37 wild type strain.

### Time lapse microcinematographies

Samples of mid-log phase cultures of G37 wild type strain and Δmg491-mg491cat, Δmg491-mg491C87S, Δmg491-mg491F157A-F158A and Δmg491-mg491loopL2 were 200x diluted and grown overnight on 8-well μ-slides ibiTreat (IBIDI). A Δmg491-mg491ΔNt undiluted sample was also grown overnight on 8-well μ-slides ibiTreat. Culture medium was replaced with fresh pre-warmed SP-4 before observations. Cell motility was examined at 37°C and 5% (v/v) CO_2_ using a Nikon Eclipse TE 2000-E inverted microscope. Images were captured at 2 sec intervals for 2 min. The percentage of motile cells in each strain was measured from 200 single cells and the differences were considered significant when the P value <0.05 using a standard χ² test. The mean velocity was measured from 25 motile cells of each strain and a significant difference was considered to be a P value <0.05 using a standard T-test.

### Phase contrast and epifluorescence microscopy

A sample of mid-log phase culture of mg491-F157A-F158A strain was diluted 200x in SP-4 and grown overnight on 8-well μ-slides ibiTreat (IBIDI). Just before visualizing cells, culture medium was replaced with fresh pre-warmed SP-4 containing Hoechst 33342 0.01 mg/ml. Cells were observed by phase contrast and epifluorescence in a Nikon Eclipse TE 2000-E inverted microscope. Phase contrast and DAPI (excitation 387/11 nm, emission 447/60 nm) epifluorescence pictures were captured with a Digital Sight DS-SMC Nikon camera controlled by NIS-Elements BR software.

### Scanning electron microscopy

Samples of *M*. *genitalium* G37 wild type and mutant strains were diluted as previously described and grown overnight in SP-4 medium over coverslips at 37°C and 5% (v/v) CO2. Then, coverslips were dehydrated and metalized as previously described [17] and were visualized in a Merlin scanning electron microscope (Zeiss). The percentage of single cells with more than one terminal organelle and the percentage of cells with a size smaller than 0.35 μm were measured from 200 single cells. A significant difference was considered to be a P value <0.05 using a χ² test.

### Cryo-electron microscopy

Samples of *M*. *genitalium* G37 wild type and mutant strains were diluted as previously described and grown overnight in SP-4 medium over holey carbon-coated grids at 37°C and 5% (v/v) CO_2_. Each grid was washed with 1x PBS supplemented with 0.9 mM CaCl_2_ and 0.49 mM MgCl_2_ (PBSCM, Sigma), blotted to remove the liquid excess and immediately plunged into liquid ethane in a Leica EM CPC cryo-workstation (Leica Microsystems). The grids were transferred to liquid nitrogen and kept at -179°C during image capturing in a 626 Gatan cryoholder (Gatan). The grids were examined on a JEOL 2011 transmission electron microscope operating at an accelerating voltage of 200 kV. Micrographs were recorded using a Gatan USC1000 camera under low electron dose conditions to minimize damage by electron beam radiation. A moderate underfocus between -30 μm and -15 μm was used to increase the contrast of the micrographs.

## Supporting Information

S1 Fig Harker section of native Patterson maps for different data sets. The clear pseudo origin peak (in the left) corresponding to the two-fold NCS axis exhibited very significant variations for different data sets. These differences correspond to variations in the orientation of the symmetry axis, which also reflects the low isomorphism of the data sets.
(TIFF)

S2 Fig Sample of the experimental electron density maps. Stereo view of the electron density map showing the interdigitation of helices in the subunits core. The 2Fo-Fc map is contoured at 1.5 σ.
(TIFF)

S3 Fig Oligomerization analysis. (a) Chromatographic profiles of MG491 (blue) and MG491-Nt (red) proteins in a calibrated Superdex 200 16/60 gel filtration column. The approximate apparent molecular weight is indicated and would be adequate for a tetramer. (b) SDS-PAGE analysis of glutaraldehyde-induced crosslinking showing the presence of dimers and larger aggregates of MG491 and MG491-F157A-F158A proteins, having a very similar behavior [38]. Lane M, indicates molecular weight standards. (c) Mass spectrum (acquisition range: 500–8000 m/z) of 10 μM MG491-Nt in a 100 mM NH4OAc buffer solution. The m/z ions corresponding to the monomer are the predominant species, but the tetramer is also detected at m/z 4968 (29+) and 4802 (30+) (red arrows).
(TIF)

S4 Fig Symmetric oligomers. Oligomerization according to the tight interface would result in a right-handed helical aggregate containing at most four subunits (top). Oligomerization according to the loose interface would result in a left-handed helical aggregate with only three subunits (bottom).
(TIFF)

S5 Fig Interactions involving loop L2 residues. LigPlot^+^ diagrams showing the inter- and intra-molecular interactions in the (a) tight dimer and (b) loose dimer. Despite the shift of Phe157 and Phe158 residues toward the neighbor monomer within the loose dimer (see also Fig 4A and 4B), the pairing of Phe157 and a Gly residue (Gly80 and Gly91 in the tight and loose dimer, respectively) is preserved. The central part of loop L2 (Leu154-Asn159) is represented in grey in both panels. Glycine residue interacting with Phe157 in each dimer is boxed in bright green. Carbon, nitrogen and oxygen atoms are colored as black, blue and red, respectively. Hydrophobic interactions are represented by grey semicircles with radial spokes, while hydrogen bonds are shown as green dotted lines with their lengths in angstroms.
(TIFF)

S6 Fig Hemadsorption activity of *M*. *genitalium* G37 wild type and MG491 mutant strains. A fixed amount of cells from each mycoplasma strain was mixed with increasing concentrations of red blood cells. The fraction of free mycoplasma cells was detected by flow cytometry and fitted to inverse Langmuir Isotherms as described previously [63]. The dissociation constant (K_D_) and the maximum fraction of mycoplasma attached to red blood cells (B_max_) was determined for each strain by iteration and are shown in the table. The Δmg491 and mg491-ΔNt mutant strains exhibited a non-hemadsorption phenotype similar to that exhibited by mg491^-^ mutant strain [63] and could not be properly fitted to an inverse Langmuir Isotherm. The binding parameters from mg491-mg491cat and mg491-C87S mutant strains showed no statistically significant differences with G37 wild type strain, indicating that the hemadsorption in Δmg491 cells was restored upon the introduction of a mg491 wild type allele or the mg491C87S mutant allele by transposition. Finally, the K_D_ from mg491-F157A-F158A and mg491-ΔloopL2 mutant strains were significantly higher than the K_D_ from G37 wild type strain, indicating that these strains have an intermediate hemadsorption phenotype and showing that these alleles could only partially complement the hemadsorption deficiencies in the Δmg491 mutant strain.
(TIF)

S7 Fig Phase contrast and epifluorescence microscopy of minute cells from the mg491-F157A-F158A strain. To test the presence of DNA in the motile minute cells from the mg491-F157A-F158A strain, these cells were stained with Hoechst 33342, examined by time lapse microcinematography and finally visualized by epifluorescence microscopy. First, 105 minute cells were identified by their size in the different microcinematographies. Most of the minute cells analyzed (93.3%) showed no detectable fluorescence after staining with Hoechst indicating that these cells did not contain detectable amounts of DNA (white arrows). Among these non-fluorescent cells, 53 of them (54.1%) were found motile during the examination period (S4 Movie). These results are in agreement with previous works suggesting that minute cells are, in fact, DNA-free terminal organelles detached from the main cell body [64,65]. Bar is 10 μm.
(TIF)

S8 Fig Intrinsic fluorescence coupled with static light scattering measurements. Temperature dependence of fluorescence and static light scattering for both wild type MG491 and variant Phe157Ala-Phe158Ala. Conformational stability and aggregation propensity of each sample is estimated by monitoring changes in fluorescence and light scattering, respectively. The experiment was performed with 9 μl of the wild type or the variant protein, which were loaded at two different concentrations (in 0.02 M Tris-HCl buffer (pH 8) containing 0.15 M NaCl) and analyzed in duplicate on the Optim 1000 (Avacta Group plc). A linear temperature ramp was applied between 15 and 90°C at a rate of 1°C/min. (a) Evaluation of aggregation propensity (obtained by recording changes in light scattering intensity at 266 nm). (b) The barycentric fluorescence, which represents the wavelength at weighted maximum of intrinsic fluorescence. It can be concluded that proteins from the wild type and the variant Phe157Ala-PheF158Ala are thermally stable, as aggregation starts to occur at T ≥ 55°C, with a clear a shift in the apparent aggregation onset temperature between the two samples, while the unfolding behavior appears unaffected.
(TIF)

S1 Movie Time-lapse microcinematography of *M*. *genitalium* G37 wild type cells. Bar is 5 μm.
(AVI)

S2 Movie Time-lapse microcinematography of *M*. *genitalium* Δmg491-mg491cat cells. Bar is 5 μm.
(AVI)

S3 Movie Time-lapse microcinematography of *M*. *genitalium* mg491-C87S cells. Bar is 5 μm.
(AVI)

S4 Movie Time-lapse microcinematography of *M*. *genitalium* mg491-ΔloopL2 cells. Bar is 5 μm.
(AVI)

S5 Movie Time-lapse microcinematography of *M*. *genitalium* mg491-F157A-F158A cells. Red arrows point to motile minute cells showing no Hoechst fluorescence in S7 Fig. Blue arrow points to a non-motile minute cell showing no Hoechst fluorescence in S7 Fig. Bar is 5 μm.
(AVI)

S6 Movie Time-lapse microcinematography of *M*. *genitalium* mg491-ΔNt cells. Bar is 5 μm.
(AVI)

S1 Table Correspondences between *M*. *genitalium* and *M*. *pneumoniae* orthologs. (DOC)

S2 Table Oligonucleotides used in this work. (DOC)
