# Supplementary figures and images for: Structure-Guided Mutations in the Terminal Organelle Protein MG491 Cause Major Motility and Morphologic Alterations on Mycoplasma genitalium
Source: PLoS Pathog. 2016 Apr 15;12(4):e1005533. doi: 10.1371/journal.ppat.1005533 (PMC4833410; doi:10.1371/journal.ppat.1005533)

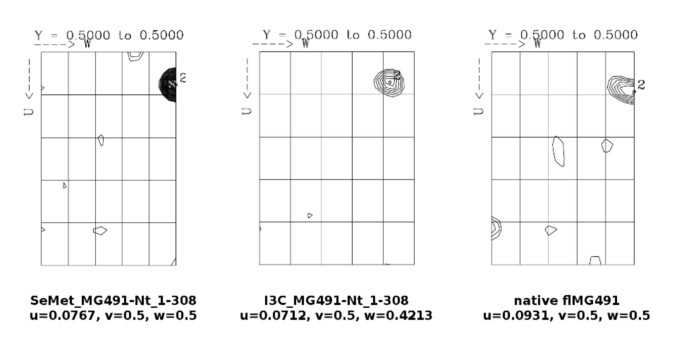

Supplement: S1 Fig — The clear pseudo origin peak (in the left) corresponding to the two-fold NCS axis exhibited very significant variations for different data sets. These differences correspond to variations in the orientation of the symmetry axis, which also reflects the low isomorphism of the data sets. (TIFF) [file ppat.1005533.s001.tiff]

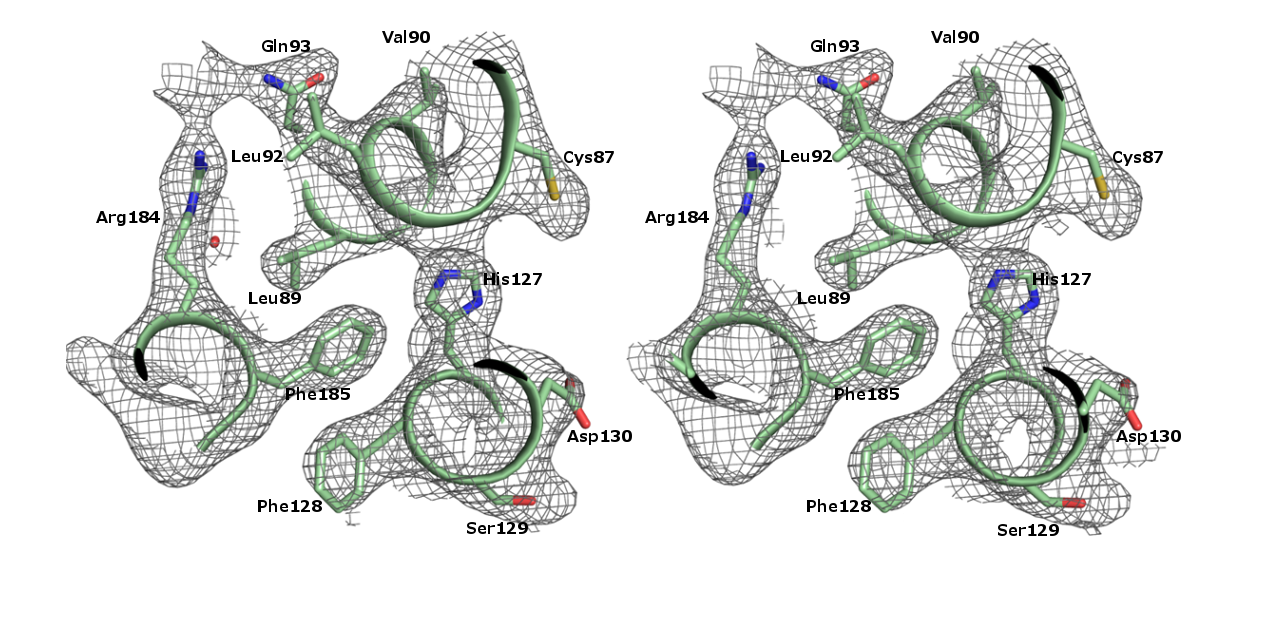

Supplement: S2 Fig — Stereo view of the electron density map showing the interdigitation of helices in the subunits core. The 2Fo-Fc map is contoured at 1.5 σ. (TIFF) [file ppat.1005533.s002.tiff]

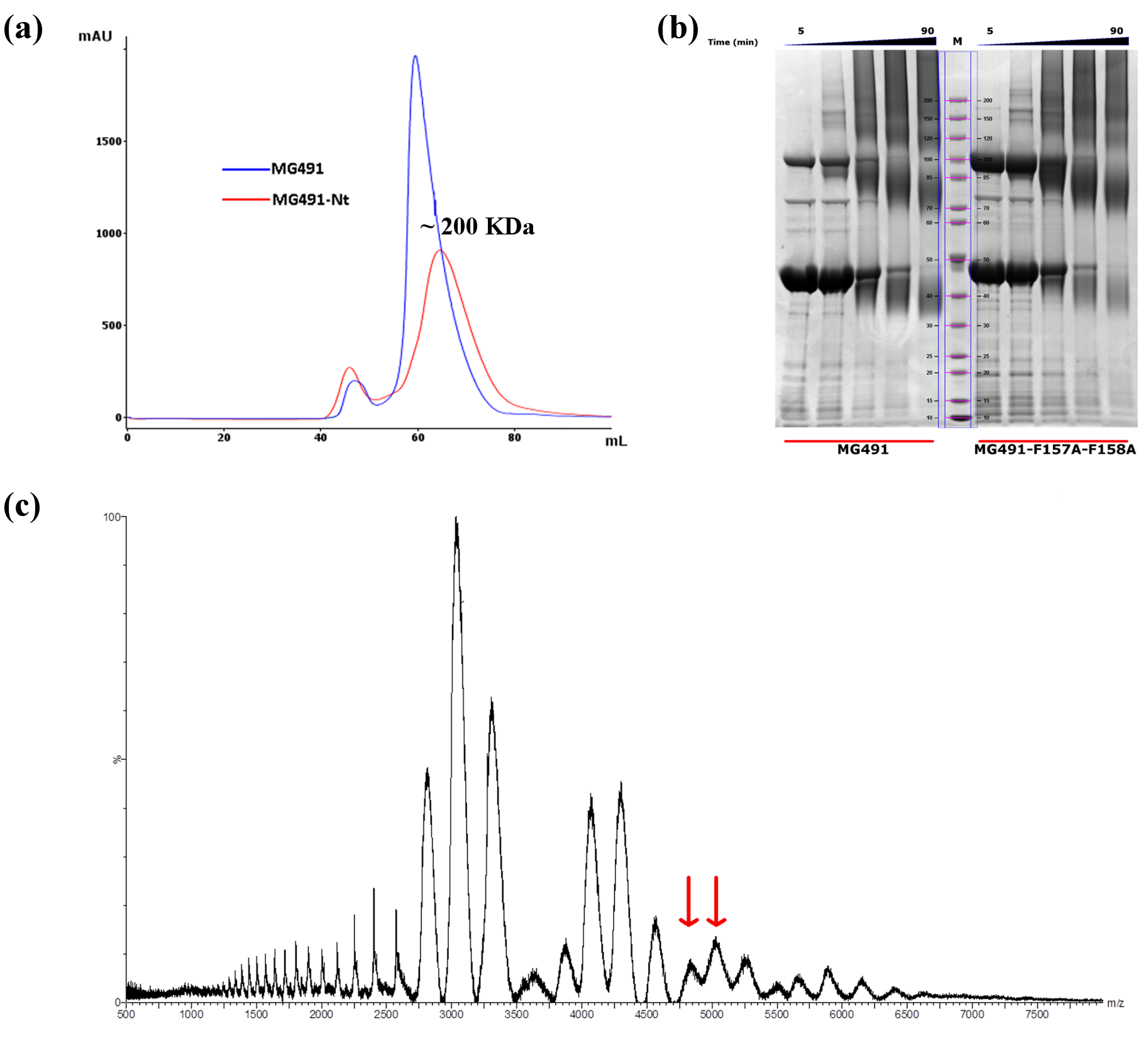

Supplement: S3 Fig — (a) Chromatographic profiles of MG491 (blue) and MG491-Nt (red) proteins in a calibrated Superdex 200 16/60 gel filtration column. The approximate apparent molecular weight is indicated and would be adequate for a tetramer. (b) SDS-PAGE analysis of glutaraldehyde-induced crosslinking showing the presence of dimers and larger aggregates of MG491 and MG491-F157A-F158A proteins, having a very similar behavior [38]. Lane M, indicates molecular weight standards. (c) Mass spectrum (acquisition range: 500–8000 m/z) of 10 μM MG491-Nt in a 100 mM NH4OAc buffer solution. The m/z ions corresponding to the monomer are the predominant species, but the tetramer is also detected at m/z 4968 (29+) and 4802 (30+) (red arrows). (TIF) [file ppat.1005533.s003.tif]

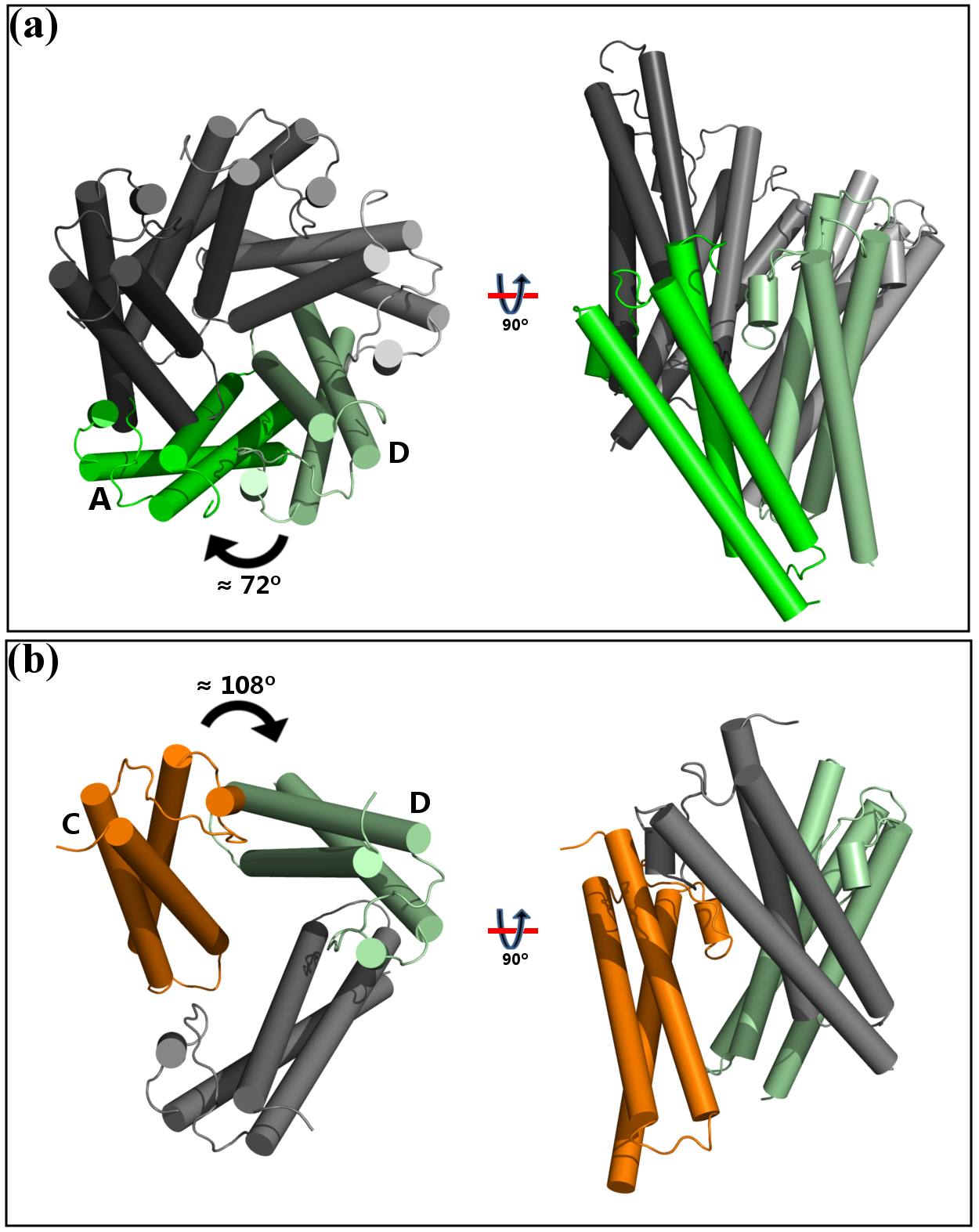

Supplement: S4 Fig — Oligomerization according to the tight interface would result in a right-handed helical aggregate containing at most four subunits (top). Oligomerization according to the loose interface would result in a left-handed helical aggregate with only three subunits (bottom). (TIFF) [file ppat.1005533.s004.tiff]

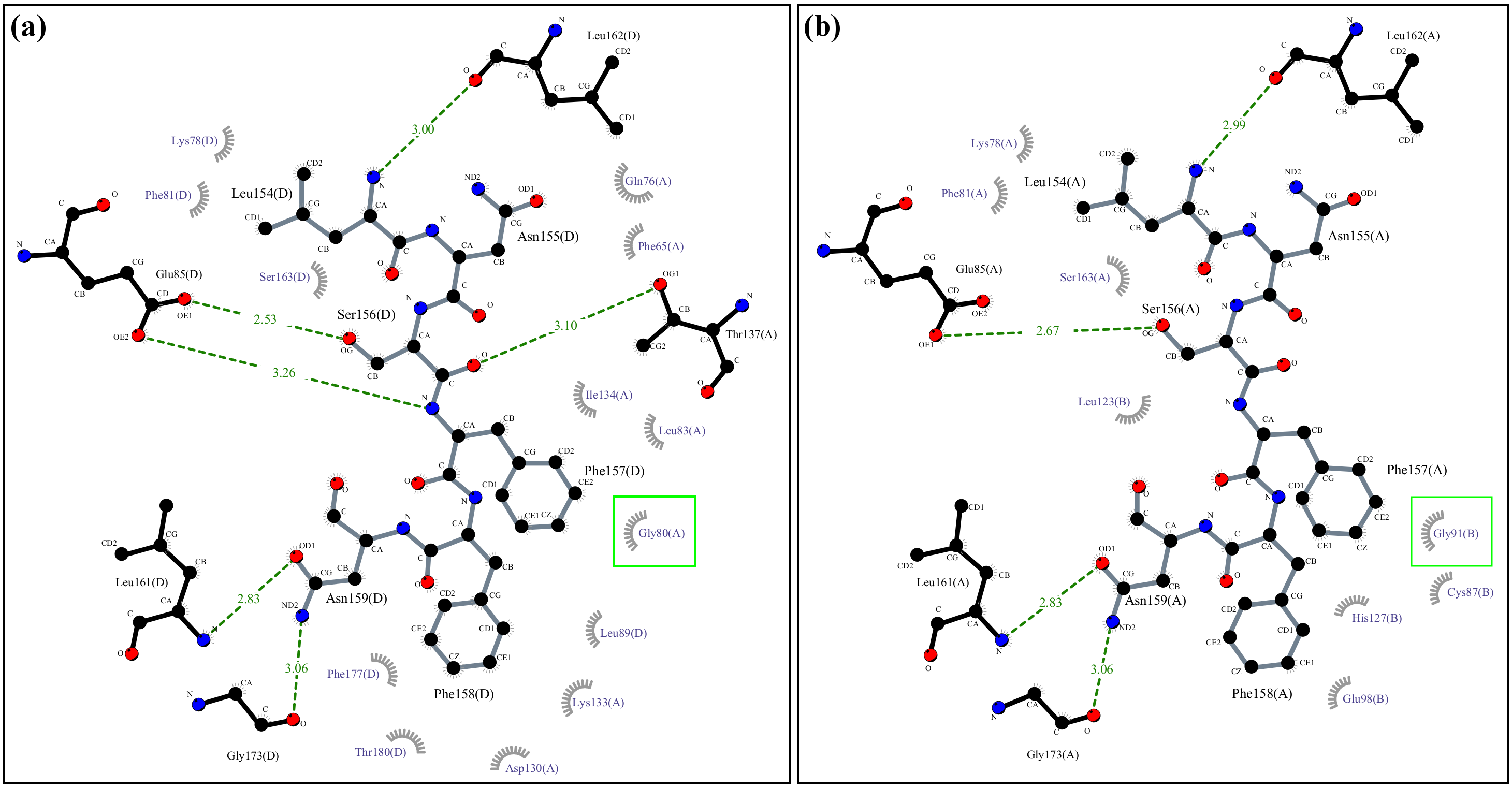

Supplement: S5 Fig — LigPlot+ diagrams showing the inter- and intra-molecular interactions in the (a) tight dimer and (b) loose dimer. Despite the shift of Phe157 and Phe158 residues toward the neighbor monomer within the loose dimer (see also Fig 4A and 4B), the pairing of Phe157 and a Gly residue (Gly80 and Gly91 in the tight and loose dimer, respectively) is preserved. The central part of loop L2 (Leu154-Asn159) is represented in grey in both panels. Glycine residue interacting with Phe157 in each dimer is boxed in bright green. Carbon, nitrogen and oxygen atoms are colored as black, blue and red, respectively. Hydrophobic interactions are represented by grey semicircles with radial spokes, while hydrogen bonds are shown as green dotted lines with their lengths in angstroms. (TIFF) [file ppat.1005533.s005.tiff]

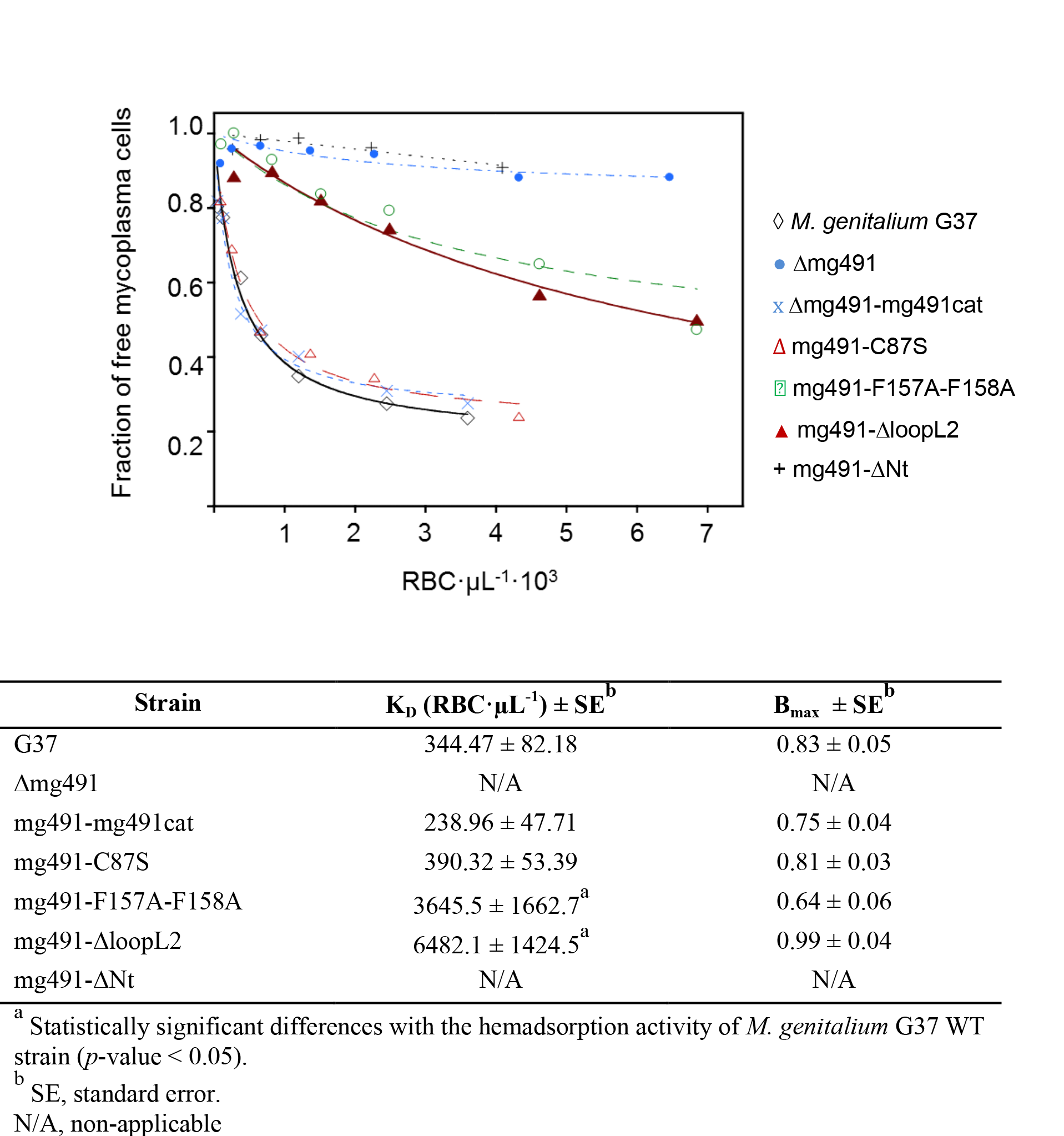

Supplement: S6 Fig — A fixed amount of cells from each mycoplasma strain was mixed with increasing concentrations of red blood cells. The fraction of free mycoplasma cells was detected by flow cytometry and fitted to inverse Langmuir Isotherms as described previously [63]. The dissociation constant (KD) and the maximum fraction of mycoplasma attached to red blood cells (Bmax) was determined for each strain by iteration and are shown in the table. The Δmg491 and mg491-ΔNt mutant strains exhibited a non-hemadsorption phenotype similar to that exhibited by mg491- mutant strain [63] and could not be properly fitted to an inverse Langmuir Isotherm. The binding parameters from mg491-mg491cat and mg491-C87S mutant strains showed no statistically significant differences with G37 wild type strain, indicating that the hemadsorption in Δmg491 cells was restored upon the introduction of a mg491 wild type allele or the mg491C87S mutant allele by transposition. Finally, the KD from mg491-F157A-F158A and mg491-ΔloopL2 mutant strains were significantly higher than the KD from G37 wild type strain, indicating that these strains have an intermediate hemadsorption phenotype and showing that these alleles could only partially complement the hemadsorption deficiencies in the Δmg491 mutant strain. (TIF) [file ppat.1005533.s006.tif]

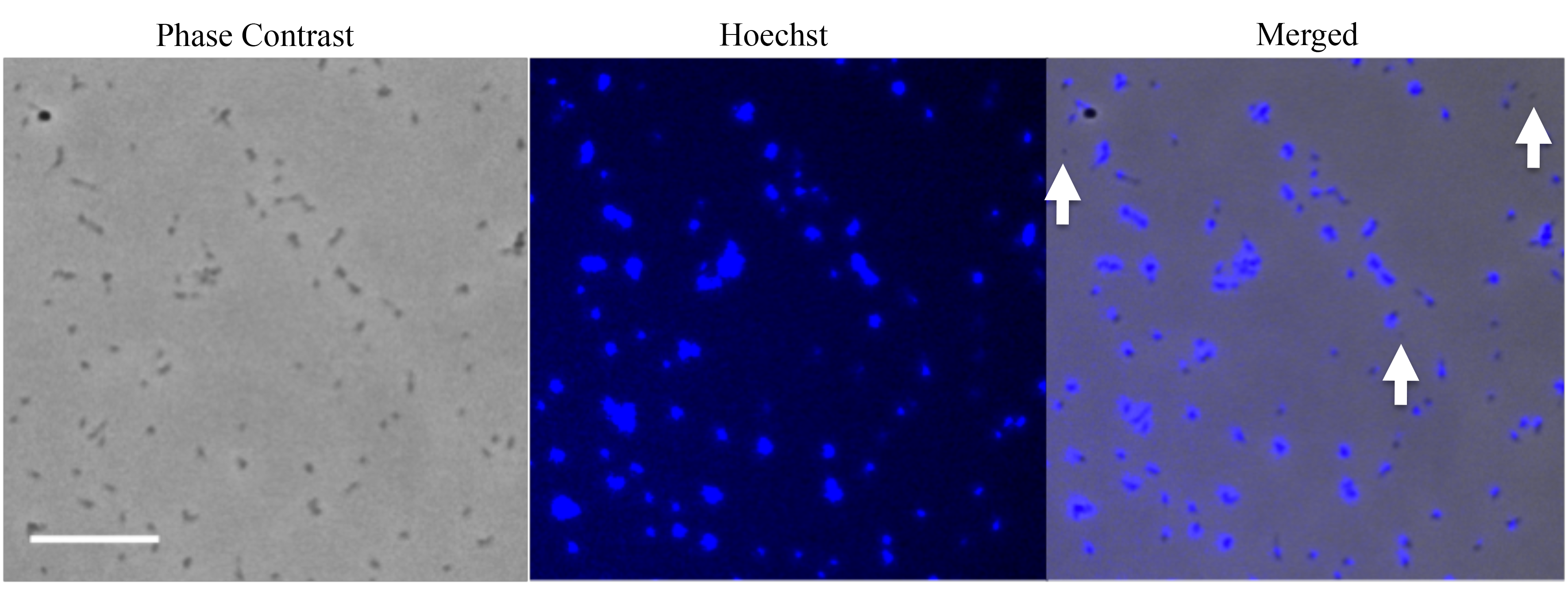

Supplement: S7 Fig — To test the presence of DNA in the motile minute cells from the mg491-F157A-F158A strain, these cells were stained with Hoechst 33342, examined by time lapse microcinematography and finally visualized by epifluorescence microscopy. First, 105 minute cells were identified by their size in the different microcinematographies. Most of the minute cells analyzed (93.3%) showed no detectable fluorescence after staining with Hoechst indicating that these cells did not contain detectable amounts of DNA (white arrows). Among these non-fluorescent cells, 53 of them (54.1%) were found motile during the examination period (S4 Movie). These results are in agreement with previous works suggesting that minute cells are, in fact, DNA-free terminal organelles detached from the main cell body [64,65]. Bar is 10 μm. (TIF) [file ppat.1005533.s007.tif]

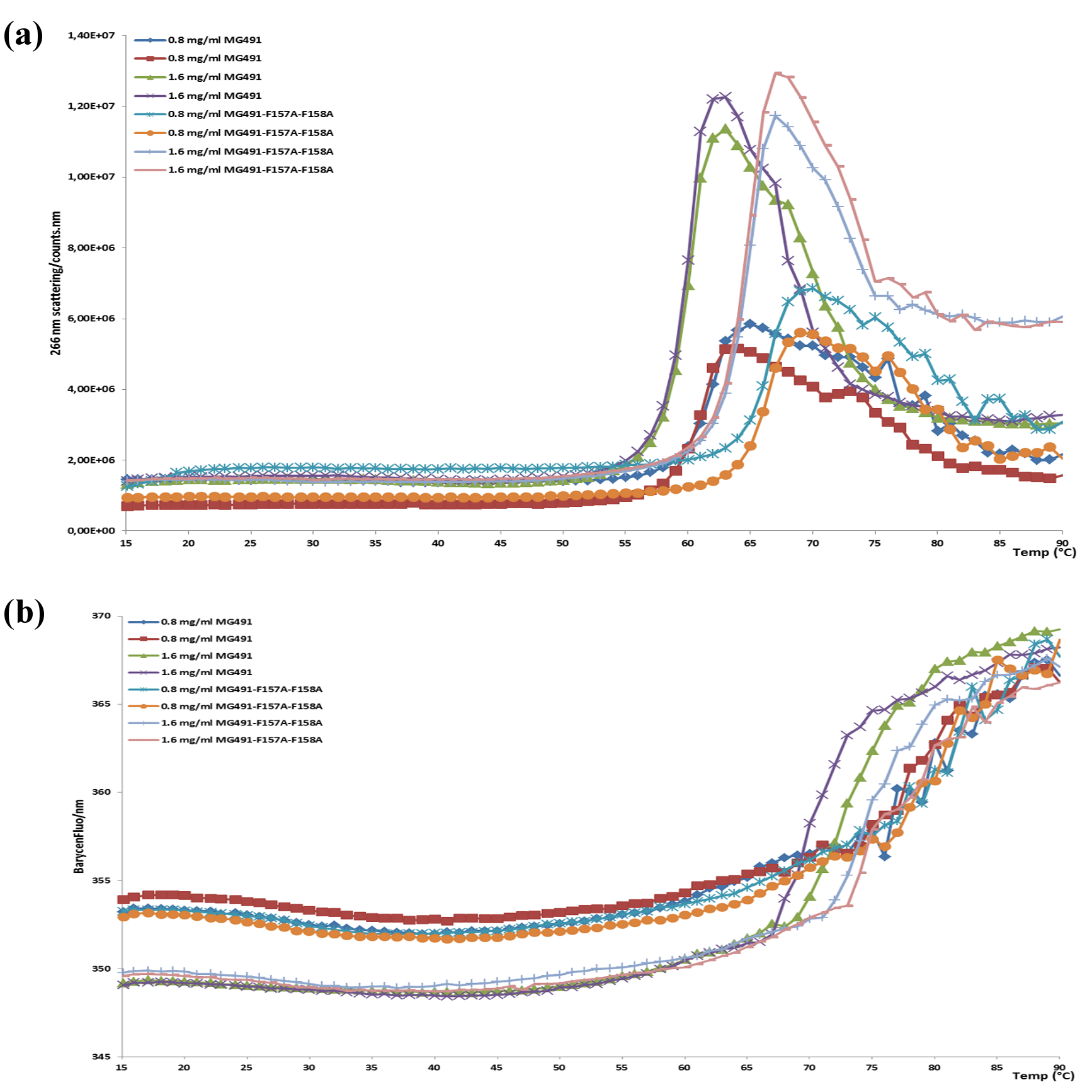

Supplement: S8 Fig — Temperature dependence of fluorescence and static light scattering for both wild type MG491 and variant Phe157Ala-Phe158Ala. Conformational stability and aggregation propensity of each sample is estimated by monitoring changes in fluorescence and light scattering, respectively. The experiment was performed with 9 μl of the wild type or the variant protein, which were loaded at two different concentrations (in 0.02 M Tris-HCl buffer (pH 8) containing 0.15 M NaCl) and analyzed in duplicate on the Optim 1000 (Avacta Group plc). A linear temperature ramp was applied between 15 and 90°C at a rate of 1°C/min. (a) Evaluation of aggregation propensity (obtained by recording changes in light scattering intensity at 266 nm). (b) The barycentric fluorescence, which represents the wavelength at weighted maximum of intrinsic fluorescence. It can be concluded that proteins from the wild type and the variant Phe157Ala-PheF158Ala are thermally stable, as aggregation starts to occur at T ≥ 55°C, with a clear a shift in the apparent aggregation onset temperature between the two samples, while the unfolding behavior appears unaffected. (TIF) [file ppat.1005533.s008.tif]
