# Supplementary material for: Structure-Guided Mutations in the Terminal Organelle Protein MG491 Cause Major Motility and Morphologic Alterations on Mycoplasma genitalium
Source: PLoS Pathog. 2016 Apr 15;12(4):e1005533. doi: 10.1371/journal.ppat.1005533 (PMC4833410; doi:10.1371/journal.ppat.1005533)
Supplement: S1 Table — (DOC) [file ppat.1005533.s015.doc]

**Table S1. Correspondences between *M. genitalium* and *M. pneumoniae* orthologs.**

| *M. genitalium* | | | | | *M. pneumoniae* | | | |
| --- | --- | --- | --- | --- | --- | --- | --- | --- |
| Locus Tag^a^ | Locus Tag^b^ | Protein | Accession nr. | Role (Reference) |  | Locus Tag^c^ | Protein | Accession nr. |
| MG_191/mgpB | MG_RS01075 | P140 | WP_010869366.1 | Adhesion (Burgos *et al*.. 2006) |  | MPN141 | P1 | NP_109829.1 |
| MG_192/mgpC | MG_RS01080 | P110 | WP_041593683.1 | Adhesion (Bugos *et al*., 2006) |  | MPN142^d^ | P40/P90 | NP_109830.1 |
| MG_200 | MG_RS01130 | MG200 | WP_010869369.1 | Gliding (Pich *et al.*, 2006) |  | MPN119 | TopJ | NP_109807.1 |
| MG_217 | MG_RS01275 | MG217 | WP_010869376.1 | Gliding (Burgos *et al*., 2008) |  | MPN309 | P65 | NP_109997.1 |
| MG_218 | MG_RS01280 | MG218 | WP_010869377.1 | Gliding/Adhesion (Pich *et al*., 2008) |  | MPN310 | HMW2 | NP_109998.1 |
| MG_219 | MG_RS01285 | MG219 | WP_010869378.1 | Unknown |  | -^e^ | - |  |
| MG_312 | MG_RS01865 | MG312 | WP_010869424.1 | Gliding/Adhesion (Burgos *et al*., 2007) |  | MPN447 | HMW1 | NP_110135.1 |
| MG_317 | MG_RS01890 | MG317 | WP_010869426.1 | Gliding/Adhesion (Pich *et al*., 2008) |  | MPN452 | HMW3 | NP_110140.1 |
| MG_318 | MG_RS01895 | P32 | WP_010869427.1 | Adhesion (Reddy *et al.*, 1995) |  | MPN453 | P30 | NP_110141.1 |
| MG_386 | MG_RS02355 | MG386 | WP_010869461.1 | Gliding (Pich *et al.*, 2006) |  | MPN567 | P200 | NP_110256.1 |
| MG_491^f^ | MG_RS01285 | MG491 | WP_010869378.1 | Unknown |  | MPN311 | P41 | NP_109999.1 |

^a^ Names according to the *M. genitalium* G37 complete genome sequence with accession number L43967.2

^b^ Names according to the *M. genitalium* G37 complete genome sequence with accession number NC_000908.2

^c^ Names according to the *M. pneumoniae* M129 complete genome sequence with accession number NC_000912.1

^d^ Protein product of MPN142 is post-translationally cleaved in *M. pneumoniae*.

^e^ MG_219 gene shares no significant sequence identity with any *M. pneumonie* ORF and no orthologous gene could be assigned.

^f^ This locus is also known as MG_218.1.
