# Supplementary material for: Structure-Guided Mutations in the Terminal Organelle Protein MG491 Cause Major Motility and Morphologic Alterations on Mycoplasma genitalium
Source: PLoS Pathog. 2016 Apr 15;12(4):e1005533. doi: 10.1371/journal.ppat.1005533 (PMC4833410; doi:10.1371/journal.ppat.1005533)
Supplement: S2 Table — (DOC) [file ppat.1005533.s016.doc]

**Table S2. Oligonucleotides used in this work.**

| **Name** | **Sequence** |
| --- | --- |
| 5MG491 | ATTGCATATGGTTAATAATGAATATCAACAAC |
| 3MG491 | ATTGCTCGAGTTCATTATGGGTATTTTTTTCAAG |
| PECCF408-rev | GTGATGGTGATGTTTtgctaataaagcagcttccaattcattttc |
| PECCF409-rev | GTGATGGTGATGTTTtttcttctgttgttttttcaaatcagtagg |
| MutMG491PA | P-ACCTGAACTGGCCTGATAGTG |
| MutMG491PB | TTAACTCATGATCATTAGCAATATC |
| I36M_F | GAACTAAAGCAAATGCTTGTTTCAC |
| I36M_R | CTTGATTTCGTTTACGAACAAAGTG |
| I168M_F | TGATCCAATGCAACGGG |
| I168M_R | ACTAGGTTACGTTGCCC |
| I205M_F | GGCTAAAATGGCCACTG |
| I205M_R | CCGATTTTACCGGTGAC |
| I313M_F | CTTAACTTTATGACCCGTCC |
| I313M_R | GAATTGAAATACTGGGCAGG |
| CmDown | CAGTACTGCGATGAGTGGCA |
| P-C87SMG491/5 | P-actggaacgcagttcgttggttg |
| C87SMG491/3 | AAAAGAAAACCACGTTTTGCTTG |
| FFAAMG491/5 | aactcggctgctaacaagcttttaagtgatc |
| P-loop/3 | P-taagacaggggtatattcactac |
| loop/5 | cttttaagtgatcctgatccaatc |
| MG491pr438ct/5 | TTTGGGCCC*TAGTATTTAGAATTAATAAAGT*ATGCAATCCAGTTTCCATAAC |
| MG491/3 | ATTGCTCGAGTTATTCATTATGGGTATTTTTTTCAAG |

Restriction sites are underlined.

Sequence coding for MG_438 promoter is in italics.
